# Tri-Combination Antiretroviral Therapy Induces Dose- and Time-Dependent Disruption of Intestinal Epithelial Barrier Function and Repair Responses in Human T84 Cells †

**DOI:** 10.3390/jox16050160

**Published:** 2026-08-26

**Authors:** Yaswanthi Yanamadala, Kuppan Gokulan, Sangeeta Khare

**Affiliations:** Division of Microbiology, National Center for Toxicological Research, US Food and Drug Administration, 3900 NCTR Rd, Jefferson, AR 72079, USA; yaswanthi.yanamadala@fda.hhs.gov (Y.Y.); kuppan.gokulan@fda.hhs.gov (K.G.)

**Keywords:** antiretroviral therapy, ART toxicity, cytokines, epithelial repair, gut barrier, HIV therapy, human T84 epithelial cells, intestinal permeability, mucin, tight junctions, wound healing

## Abstract

Antiretroviral therapy (ART) is essential for controlling human immunodeficiency virus (HIV) infection, requiring strict daily adherence for lifelong viral suppression. However, this continuous oral dosing results in persistent exposure of the gastrointestinal tract (GIT), raising the need to investigate the effects of TC-ART (Tri-combinationL: Abacavir, Dolutegravir, Lamivudine–ART) on epithelial integrity, barrier recovery mechanisms, and surface barrier architecture. TC-ART exposure (125 µM to 4000 µM) showed marked alterations in transepithelial resistance, permeability, and wound-healing abilities even at sub-cytotoxic doses. The dose exposure range at the mid-dose level showed the highest transcriptional activity, characterized by a downregulation of junctional genes [claudins (*CLDNs*), desmogleins (*DSGs*), and junctional plakoglobin (*JUP*)] and signaling mediators [the signal transducer and activator of transcription 3 (*STAT3*), mitogen-activated protein kinase 1 and 3 (*MAPK1/3*), and catenin beta 1 (*CTNNB1*)], along with reduced *IL-9* expression that is linked to mucin loss. These transcriptional changes were consistent with structural findings, including partial transepithelial electrical resistance (TEER) recovery followed by a decline, delayed wound closure, and waning of the apical mucin layer in a dose-dependent manner. However, several cytokines, like IL-2 and IL-6, showed increased secretion despite lower transcriptional levels, suggesting alternative regulatory control during early stress responses. Together, these results support that TC-ART exposure alters epithelial responses in a way that may transition from early adaptation to signs of impaired recovery, leading to a gradual decline in mucosal barrier function. Such concentration- and time-dependent epithelial stress may contribute to gastrointestinal disturbances observed in treated HIV populations, emphasizing the need for incorporating intestinal epithelial health endpoints in drug safety evaluations.

## 1. Introduction

The intestinal epithelium is a crucial interface for nutrient absorption and ion regulation, while simultaneously serving as a dynamic barrier for microbial, dietary, and pharmacologic exposures. Disruption of the gastrointestinal epithelial integrity can increase permeability, trigger inflammatory signaling, alter the mucin layer, and impair wound healing responses, which could further promote microbial translocation, immune activation and progressive tissue injury [1]. The Tri-combination Anti-retroviral therapy (TC-ART), consisting of the nucleoside reverse transcriptase inhibitors (NRTIs) abacavir and lamivudine and the integrase strand transfer inhibitor (INSTI) dolutegravir, is used as a first-line regimen in HIV patients due to its high efficacy, convenience, and favorable resistance profiles [2,3]. Although the systemic safety of TC-ART is well documented, its potential off-target effects on the gastrointestinal epithelium remain poorly understood. Notably, pre-clinical toxicological studies of repeated doses of dolutegravir have shown the gastrointestinal tract to be the major target organ [2]. Given that TC-ART is primarily absorbed from the small intestine, the intestinal epithelium is directly exposed to the drug at both luminal and mucosal surfaces [4,5], where these repeated exposures could disrupt epithelial homeostasis.

People living with HIV exhibit persistent epithelial problems [6] even with effective ART and viral suppression, including increased permeability [7] and microbial translocation [8]. These persistent abnormalities align with evidence showing that the gastrointestinal tract is one of the earliest and most profoundly affected anatomical and immunological compartments to external physiological and pharmacological stressors [2]. Furthermore, people living with HIV remain at high risk for gastrointestinal complications [9], including mucosal damage, ulcers, GERD, and chronic immune activation (leaky gut), highlighting the importance of intestinal epithelial integrity and health in long-term clinical outcomes. While the gastrointestinal consequences of HIV infection and persistent immune activation have been extensively investigated, the direct toxicological effects of TC-ART on intestinal epithelial function are poorly understood.

Our earlier in vivo works explored this question from a developmental context [10,11,12]. In these studies, second-generation rat offspring exposed to TC-ART through maternal administration during gestation and lactation exhibited sex-specific alterations in intestinal permeability, cytokine profiles, and mucosa-associated microbial populations, as well as fecal microbial abundance [10,11,12]. Although the offspring received indirect and comparatively lower systemic exposures, we observed significant alterations in the offspring intestinal structure and immune signaling, suggesting that even minimal developmental exposures to TC-ART can influence gut functioning and development. These findings raise the possibility that the intestinal epithelium itself could be the key locus for drug-induced vulnerabilities. The present study builds on this foundation by focusing on the intestinal epithelial cells.

In this study, the human colonic epithelial cell line T84 was used for investigating the dose- and time-dependent effects of TC-ART (125–4000 µM) on key epithelial function. T84 cells were chosen as they possess the ability to form polarized, high-resistant monolayers with microvilli and physiologically relevant tight junctions [13,14]. The assessments include: (i) transepithelial electrical resistance (TEER) and expression of a 96-gene panel encompassing tight junctions, adherens junctions and cytoskeletal regulators; (ii) wound-healing kinetics using live-imaging and corresponding gene markers of migration and restitution; (iii) quantification of cytokine and chemokine secretion using a 48-plex panel to evaluate epithelial immune responses; (iv) cytotoxicity measured by LDH release to distinguish functional effects from overt toxicity; and (v) mucin secretion and distribution analyzed by immunohistochemistry to assess alterations in the mucosal protective layer. We hypothesize that even at sub-cytotoxic concentrations, TC-ART may compromise epithelial barrier integrity, delay wound restitution, alter cytokine signaling, and disrupt mucin organization. Together, these effects could represent a potential mechanism through which long-term TC-ART exposures could contribute to persistent intestinal injury. By examining these responses across different doses and time points, this study aims to define how functional, transcriptional, and cytokine changes interact to reveal the epithelial vulnerability of the gut and guide future strategies to preserve barrier health during chronic therapy.

## 2. Materials and Methods

### 2.1. Dosing Rationale

The concentration range used for in vitro exposure (125–4000 µM) was selected to reflect the physiologically relevant drug levels at the intestinal surface. These doses were selected based on preliminary dose-range findings and cell viability experiments. Although the plasma *Cmax* for abacavir, lamivudine, and dolutegravir (following standard Triumeq dosing) was reported approximately at 7–15 µM per component [15], this does not represent the concentrations present at the apical epithelial surface. After oral administration, TC-ART is absorbed through the small intestine [15], where transient luminal concentrations are much higher and can reach the millimolar range prior to dilution and absorption. Following the FDA’s Drug–Drug Interaction (DDI) Guidance assumption of ~250 mL of intestinal fluid volume [16], estimated upper-bound concentrations (Dose/250 mL: abacavir ≈ 8 mM (600 mg in 250 mL); lamivudine ≈ 5 mM (300 mg in 250 mL); dolutegravir ≈ 0.5 mM (50 mg in 250 mL)) [17] were calculated. These luminal concentrations can vary depending on several factors, such as fast/fed state, intestinal fluid volume (which alters osmolality, thus changing the rate of absorption) [18,19], gastrointestinal motility and physiological conditions [17]. Based on these pharmacokinetic parameters, the selected concentrations were intended to approximate apical (luminal) exposure at the epithelial surface, while acknowledging that plasma-like basolateral exposure typically occurs within the 1–20 µM range. Furthermore, the drug in the intestinal lumen is not protein-bound (whereas dolutegravir is ~99% protein-bound in plasma [20]), and plasma Cmax values underestimate the total dissolved fraction encountered by the apical epithelium, supporting the inclusion of concentrations above plasma levels in the study. Unless stated otherwise, the reported TC-ART concentrations (125–4000 µM) represent the final combined concentration of the three-drug mixture (abacavir: lamivudine: dolutegravir), prepared at a ratio of 12:6:1.

### 2.2. Epithelial Cell Culture

Human colonic epithelial T84 cells (ATCC, CCL-248™, Manassas, VA, USA) were cultured in a humidified incubator at 37 °C, 5% CO_2_, using DMEM/F12 (Gibco) media supplemented with 10% heat-inactivated FBS (Gibco), 1% penicillin–streptomycin, and 0.2% fungizone obtained from ThermoFisher, Waltham, MA, USA. Cells were detached with trypsin (ThermoFisher, Waltham, MA, USA) and passaged once they reached 70–80% confluency. For all experiments, early passage cells (passages 5–9) were used to ensure epithelial integrity and consistent barrier properties. Cells were seeded at a density of 2 × 10^5^ cells per well in 24-well plates and maintained until a confluent monolayer was established prior to treatment. Each experiment was performed in at least three independent trials, with a minimum of three technical replicates per trial.

### 2.3. Transepithelial Electrical Resistance (TEER) Measurement

T84 epithelial cells were seeded onto collagen-coated transwell inserts (0.4 µm pore size; Corning Inc., Corning, NY, USA) and maintained under standard culture conditions (37 °C, 5% CO_2_). TEER was monitored continuously after monolayer formation (approx. 7–8 days), and cells typically became polarized around 8–10 days of culture, as indicated by stable baseline TEER values using an ECIS-TEER24 (Applied, Biophysics, Troy, NY, USA), which measures real-time impedance across gold microelectrodes in a 24-well format. TC-ART treatments (125 µM, 250 µM, 500 µM, 1000 µM, 2000 µM, and 4000 µM) were administered in the apical compartment. The TEER was continuously recorded for 48 h. Although resistance was recorded continuously, representative values were extracted and plotted at 1 h, 2 h, 3 h, 24 h, and 36 h to capture early and progressive changes in epithelial barrier function. Measurements were obtained in triplicate per concentration and normalized after subtracting background resistance from blank wells. For analysis, TEER values are expressed as percent change relative to untreated controls (set as 100%). Apical and basolateral media were collected at each time point for cytokine analysis, and the corresponding cell lysates were preserved in TRIzol™ (ThermoFisher, Waltham, MA, USA) for RNA extraction.

### 2.4. Wound-Healing Assay

T84 epithelial cells were cultured under standard conditions (37 °C, 5% CO_2_) in 24-well plates until the cells reached full confluence and a monolayer was formed [21]. A uniform scratch (mechanical wound) was made across each monolayer using a sterile 200 µL pipette tip. After gently rinsing the monolayer with media to remove any detached cells, 1 mL of a fresh culture medium containing TC-ART was added to the wells at concentrations of 125 µM, 250 µM, 500 µM, 1000 µM, 2000 µM, and 4000 µM. Cells were maintained for 14 days while replenishing with fresh media containing corresponding doses, and epithelial recovery was monitored on days 1, 3, 5, 7, 10, and 14 using phase-contrast microscopy (EVOS FL, ThermoFisher, Waltham, MA, USA). Culture supernatants at each time point were collected and stored at −80 °C for lactate dehydrogenase (LDH) quantification and cytokine measurements, while cells were preserved in TRIzol for further RNA extraction.

#### Wound-Healing Time-Kinetic Assay

After determining the concentration-dependent effects of TC-ART on wound healing, a time-dependent evaluation was conducted to study the cell viability, cytotoxicity, and transcriptional changes during the wound-healing process. Preliminary testing at higher concentrations (2000 µM and 4000 µM) resulted in early cell detachment and knockdown of several genes. Hence, intermediate concentrations of 500 µM and 1000 µM were selected for subsequent time-course experiments. A separate set of wounded T84 monolayers with similar conditions as described for the wound-healing assay were treated with TC-ART (500 µM to 4000 µM). The supernatants were collected at 1 h, 3 h, 6 h, 12 h, 24 h, and 30 h post-treatment to assess acute LDH release, and corresponding cells were stored in TRIzol reagent at −80 °C. Phase-contrast images were captured at each interval to monitor changes in wound area and closure rate, allowing for a correlation of cytotoxic response with epithelial recovery kinetics.

### 2.5. LDH Cytotoxicity Assay

Cytotoxicity was quantified using the LDH Assay Kit (TOX7; Sigma-Aldrich, St. Louis, MO, USA), according to the manufacturer’s instructions. Cell-free supernatants collected from both wound-healing and time-kinetic experiments were incubated at 37 °C, with the LDH reaction mixture (containing an assay buffer and a substrate mix) in a clear, flat-bottom 96-well plate. Absorbance was measured at 450 nm, with a reference wavelength of 680 nm, using a BioTek Cytation 5 reader (Agilent, Santa Clara, CA, USA). The percentage of LDH release was calculated relative to control (untreated) cells to determine drug-induced cytotoxicity. Data are presented as percent LDH release (mean ± SEM) of triplicate wells per treatment concentration and time points.

### 2.6. Cytokine Analysis

Apical supernatant was collected from the TEER assay and wound-healing time-kinetic assay stored at −80 °C were thawed on ice and used for cytokine analysis. Cytokine profiling was performed using a multiplex human cytokine assay panel (48-plex; Bio-Rad Laboratories, Hercules, CA, USA) following the manufacturer’s instructions. Thawed samples were centrifuged to remove cell debris and incubated with antibody-coupled magnetic beads specific to each analyte. Following sequential incubation with detection antibodies and streptavidin–phycoerythrin, fluorescence was quantified using a Bio-Plex 200 system (Bio-Rad Laboratories, Hercules, CA, USA). Concentrations were calculated from standard curves generated for each analyte, and data were normalized to controls to evaluate treatment-related inflammatory responses.

### 2.7. RNA Extraction and Gene Expression Analysis

The TRIzol-stored cell pellets collected from the TEER and wound-healing assays were subsequently used for RNA extraction. Total RNA was isolated as described previously [21], quantified using a NanoDrop spectrophotometer (ThermoFisher Scientific, Waltham, MA, USA) and assessed for purity (A260/A280 > 1.8). Genomic DNA was removed with Invitrogen DNase I (ThermoFisher Scientific, Waltham, MA, USA). Equal amounts of RNA were reverse transcribed using the SuperScript IV VILO cDNA Synthesis Kit (Invitrogen, ThermoFisher Scientific, Waltham, MA, USA). mRNA gene expression profiling of 96 epithelial-barrier-related genes or 96 wound-healing-related genes was performed using Qiagen RT^2^ Profiler PCR Arrays (Human Cell Junction Pathway Finder: PAHS-213Z; Human Wound Healing: PAHS-121Z) with the SYBR Green qPCR Master Mix (Qiagen, Germantown, MD, USA) following the manufacturer’s protocol. Reactions were run on a Bio-Rad CFX384 Real-Time PCR System (Bio-Rad, Laboratories, Hercules, CA, USA). Relative expression was calculated using the Qiagen GeneGlobe Data Analysis Center (https://geneglobe.qiagen.com/us/analyze; a web-based, cloud platform; available via Qiagen, Germantown, MD, USA), with β-actin (*ACTB*) and β-2-microglobulin (*B2M*) used as endogenous controls. Results are reported as fold-regulation relative to untreated controls (mean ± SEM, n ≥ 3 biological replicates).

### 2.8. Mucin Immunohistochemistry (IHC) and Quantification

Mucin production and distribution in T84 epithelial monolayers were evaluated following treatment with 125 µM, 500 µM, and 1000 µM concentrations of TC-ART. After 4 h and 24 h of exposure in a 24-well plate, cells were fixed with 4% paraformaldehyde for 15 min, washed with PBS, and subjected to periodic acid–Schiff’s (PAS) staining to visualize mucopolysaccharide-rich mucins. Briefly, fixed monolayers were oxidized with 0.5% periodic acid and incubated with Schiff’s reagent (Sigma-Aldrich, St. Louis, MO, USA). PAS staining was performed with or without hematoxylin counterstaining for optimal contrast of mucin-positive regions. Three randomly selected locations from each well were imaged with bright-field microscopy at 4× and 10× magnification.

Quantitative analysis of mucin coverage was performed using the Fiji/ImageJ software (Image J win 64 version 1.54p NIH, USA). Mucin-positive (magenta) regions were isolated using color thresholding with a consistent threshold value (10) across all images to ensure comparability. The percentage area of mucin staining per field was calculated and averaged from multiple randomly selected fields per sample (n = 4). Data are expressed as the mean ± SEM relative to untreated controls.

### 2.9. Statistical Analysis

Data are presented as the mean ± SEM from three independent biological experiments, each with three technical replicates, unless stated otherwise. Statistical analyses were performed using SigmaPlot version 16.x (Systat Software, San Jose, CA, USA) and Origin Pro (Origin 2023b version 10.0.5.157; Northhampton, MA, USA). Comparisons between multiple groups were analyzed using ANOVA, while comparisons between two groups were performed using an unpaired Student’s *t*-test. Bio-Plex cytokine data were analyzed using the Bio-Plex Data Pro Software version 1.2 (Bio-Rad Laboratories, Hercules, CA, USA), employing the Mann–Whitney U test for two-group comparisons and the Kruskal–Wallis test for multiple-group comparisons. RT^2^ Profiler PCR data were analyzed using Qiagen Gene Globe Data Analysis (https://geneglobe.qiagen.com/us/analyze; a web-based, cloud platform; available via Qiagen, Germantown, MD, USA) and using Student’s *t*-test on normalized values.

## 3. Results

To test our hypothesis that TC-ART may affect intestinal epithelial cells even at sub-cytotoxic concentrations, we evaluated multiple endpoints to assess intestinal-epithelial-layer health outcome. Polarized T84 monolayers grown on transwell inserts were used to assess barrier resistance, permeability-related changes, and cytokine profile whereas non-polarized monolayers cultured in 24-well plates were used to evaluate wound healing, cytokine responses, mucin production, and cytotoxicity. All functional assays were performed at sub-cytotoxic concentrations (≤1000 µM), where cell viability remained above 90%, to isolate functional barrier effects without confounding influence of cytotoxicity.

### 3.1. TC-ART Induces Early Epithelial Barrier Changes and Alters Junctional and Inflammatory Pathways

#### 3.1.1. TC-ART Causes Progressive Loss of Epithelial Resistance

**Figure 1:**
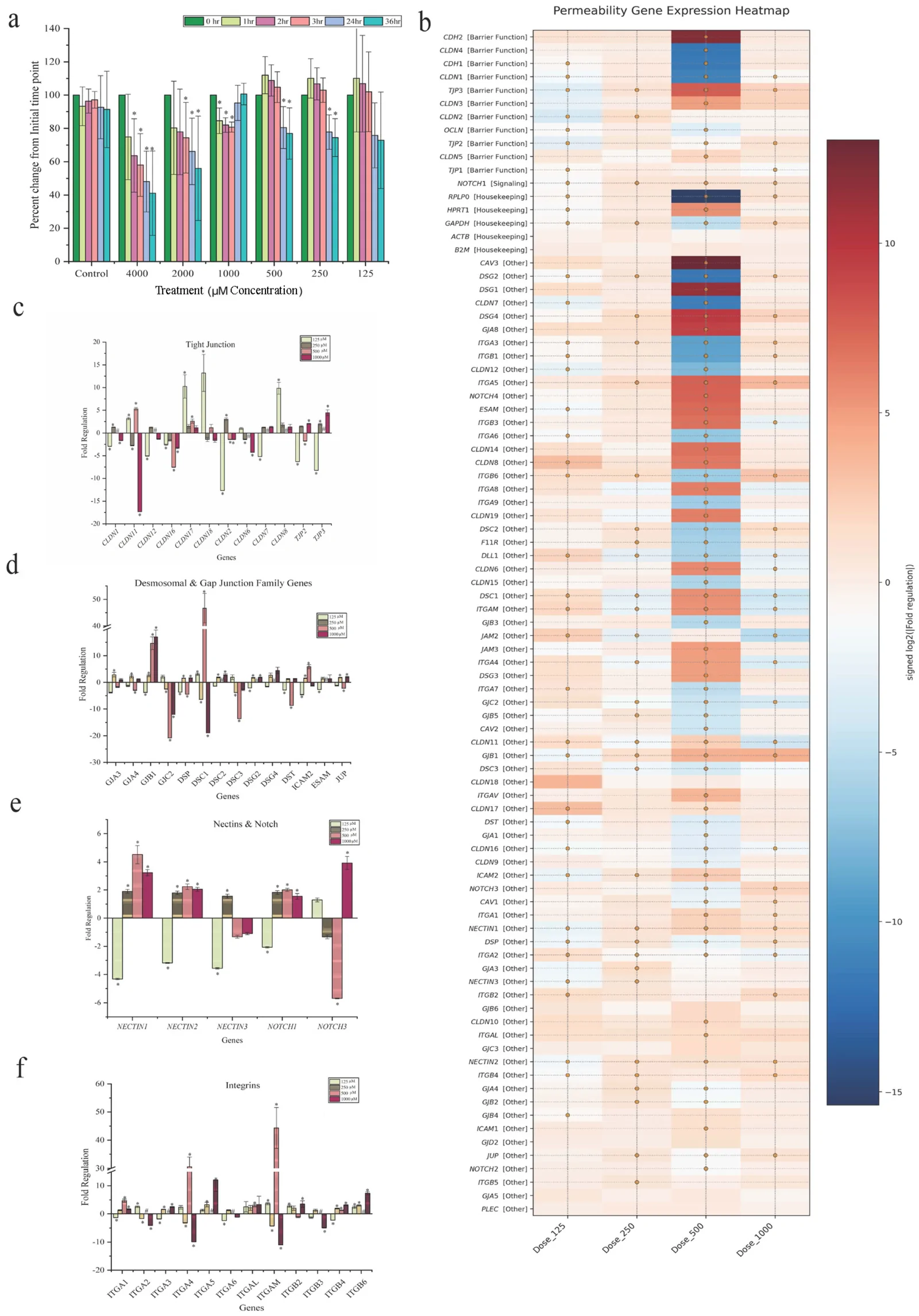
Effects of TC-ART exposure on epithelial permeability-related gene expression in T84 cells. (**a**) Transepithelial electrical resistance (TEER) measurements of T84 monolayers following exposure to increasing concentrations of TC-ART (125–4000 µM). TEER values are expressed as percent change from the initial time point and monitored over time (0–36 h). (**b**) Heatmap showing fold regulation of permeability-associated genes following drug exposure. Gene expressions were normalized against housekeeping gene. The fold regulation was calculated as compared to the untreated controls. Filled circles in the heat map represent *p* < 0.05. (**c**–**f**) Fold regulation of genes associated with (**c**) tight junctions, (**d**) desmosomal and gap junction complexes, (**e**) nectins and NOTCH signaling, and (**f**) integrin-mediated adhesion following treatment with 125–1000 µM drug concentrations. Data are presented as mean ± SEM. Statistical significance relative to control is indicated (* *p* < 0.05) (n = 3). # indicates complete knockdown like either 0 or more than 150 fold change.

To assess changes in the epithelial barrier function, TEER values were monitored following TC-ART exposure. Untreated monolayers exhibited a stable baseline resistance throughout the experiment, indicating mature tight-junction formation and epithelial polarization. In contrast, TC-ART drug exposure showed a dose- and time-dependent decrease in TEER (Figure 1a). Supraphysiological concentrations (2000 µM and 4000 µM) produced the earliest and most pronounced reductions, with decreases apparent by 2 h and a 40–50% loss by 24 h, indicating substantial barrier disruption. At 1000 µM, TEER initially declined but showed partial recovery at later time points. Lower concentrations (≤500 µM) resulted in a moderate drop in TEER values and later stabilized at approximately 70% of the baseline by the end of the monitoring period. These results indicate that TC-ART can compromise epithelial resistance even at concentrations below the cytotoxic threshold, suggesting functional tight-junction weakening prior to overt cell loss.

#### 3.1.2. Junctional Genes Show Disruption with Compensatory Activation of Adhesion Pathways

To further understand the molecular basis of TEER decline, transcriptional profiling of 96 permeability/junction-related genes, including tight junctions, adherens, desmosomal and gap junctions, and integrin families, was performed in the same cells after 48 h of TC-ART exposure. Distinct concentration-dependent changes were observed, with the most prominent fold changes occurring at 125 µM and 500 µM. The 250 µM group mostly showed the same trend as that of 125 µM and 500 µM with a smaller amplitude, while 1000 µM showed a partially dampening response (Figure 1b), consistent with the TEER data, possibly reflecting a feedback or adaptive response at higher concentrations. Among significantly altered genes, the tight-junction genes (*CLDN1*, *CLDN2*, and *CLDN16*) (Figure 1c) along with junctional and adhesion genes (*GJC2*, *DSC1*, *DSC3*, *DSG2*, *DST*, *ICAM2*, and *JUP*) (Figure 1d) were predominantly downregulated at 125 µM and 500 µM, aligning with the reduced TEER values. *CLDN17* and *CLDN8* showed an initial upregulation at 125 µM, followed by a gradual decline at higher concentrations, reflecting a non-linear, dose-dependent response.

Within the adhesion and signaling families (*NECTIN*, *NOTCH*, and integrins) the data indicate early remodeling rather than uniform suppression. *NECTIN1*, *NECTIN2*, and *NOTCH1* (Figure 1e) consistently increased across concentrations, while *NOTCH3* showed a biphasic profile. *NOTCH3* transiently reduced to near 500 µM, followed by induction at 1000 µM, which might suggest a shift from an early stress response to activation of epithelial repair pathways [22,23]. Notably, several integrins and desmocollin genes were not detected at 500 µM, indicating a marked gene suppression below the assay detection limit and suggesting a strong inhibitory effect on the adhesion pathway at this dose.

Integrins also showed a similar pattern as that of other junctional genes. Several pro-migratory integrins (*ITGA1*, *ITGA5*, and *ITGB6*—supporting epithelial spreading and restitution; *ITGB4*—anchoring integrin) were increased (Figure 1f), while the structural components (*DST* and *GJC2*) were consistently reduced across the exposure range, indicating weakened cytoskeletal and gap-junctional support despite compensatory adhesion signaling. *ITGAL*, which encodes the leukocyte integrin αL (LFA-1), was elevated. While the functional significance in epithelial cells is unclear, such induction has been reported in cellular stress and may reflect nonspecific activation of immune-associated transcription. GJB1 (connexin-32) showed a slight reduction at 125 µM, followed by a progressive increase at higher concentrations (250–1000 µM). Although a single gap-junction gene does not directly confirm functional changes, this pattern may indicate a transient low-dose suppression of connexin expression, whereas higher concentrations trigger compensatory upregulation to maintain intercellular signaling. Overall, these transcriptomic changes indicate that TC-ART predominantly disrupts pathways involved in epithelial barrier integrity and junctional remodeling in a dose-dependent manner.

#### 3.1.3. Cytokine Responses Reveal Dose-Dependent Inflammatory and Stress Signaling

**Figure 2:**
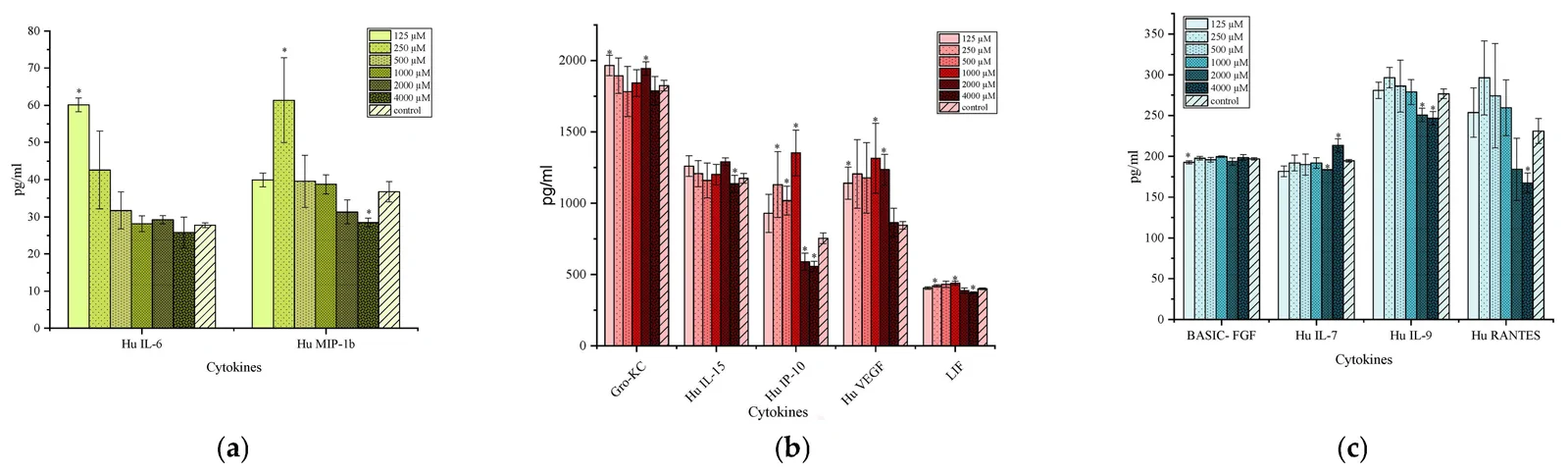
Cytokine secretion following TC-ART exposure in polarized epithelial cells from permeability assay. Cytokine concentrations measured in apical chamber media collected from polarized T84 monolayers following exposure to different concentrations of TC-ART (125–4000 µM). (**a**) Pro-inflammatory cytokines IL-6 and MIP-1β. (**b**) Chemokines and growth factors, including GRO-KC, IL-15, IP-10, VEGF, and LIF. (**c**) Additional cytokines and chemokines, including basic FGF, IL-7, IL-9, and RANTES. Cytokine levels are expressed as pg/mL. Data are presented as mean ± SEM. Statistical significance relative to control is indicated (* *p* < 0.05) (n = 3).

The supernatant from the same wells of polarized monolayers were analyzed for cytokine secretion. Lower doses, particularly 125 µM, triggered an early pro-inflammatory response, as indicated by increased IL-6 and GRO/KC levels (Figure 2a,b), with MIP-1β (250 µM) and LIF also showing a similar upward trend. These early cytokine shifts are consistent with patterns observed during epithelial stress and initiation of repair signaling (LIF and GRO/KC) [24], aligning with the TEER decline and transcriptional remodeling of junctional and integrin genes. At higher concentrations > 1000 µM, cytokines associated with repair and homeostasis (VEGF and RANTES) reduced, while anti-inflammatory IP-10 increased at low concentrations and declined at high concentrations, indicating a stressed state. Pro-inflammatory cytokines (IL-7 and IL-9) involved in epithelial homeostasis also decreased at higher concentrations (Figure 2c). Another possible explanation for reduced cytokine levels at high concentrations is decreased cellular viability or metabolic activity, which limits cytokine production despite transcriptomic activity at lower doses. Despite marked gene changes at 500 µM, cytokine levels were modest, suggesting that junctional gene responses occur earlier than secretory activation. This intermediate dose likely represents an adaptive phase where the barrier is structurally stressed but not yet producing strong cytokine signaling.

### 3.2. TC-ART Alters Mucin Secretion and Distribution in Intestinal Epithelial Monolayer Cells

**Figure 3:**
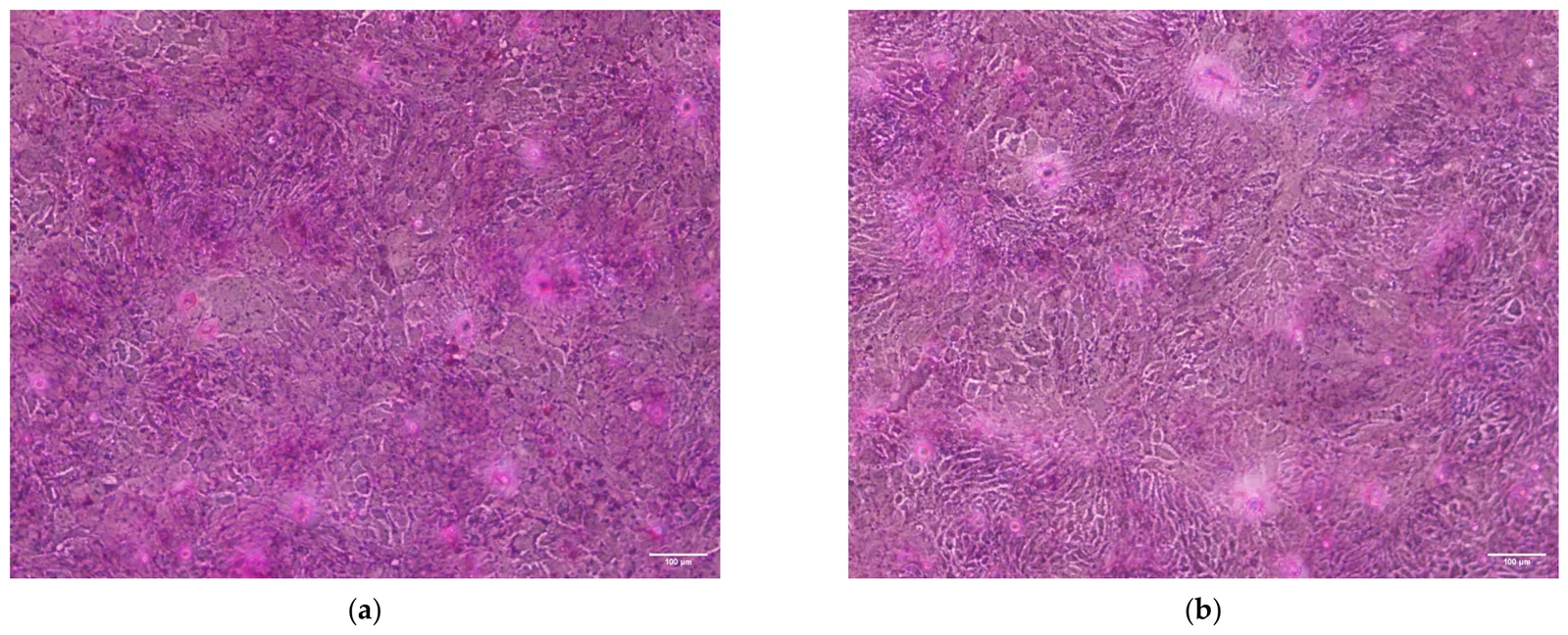

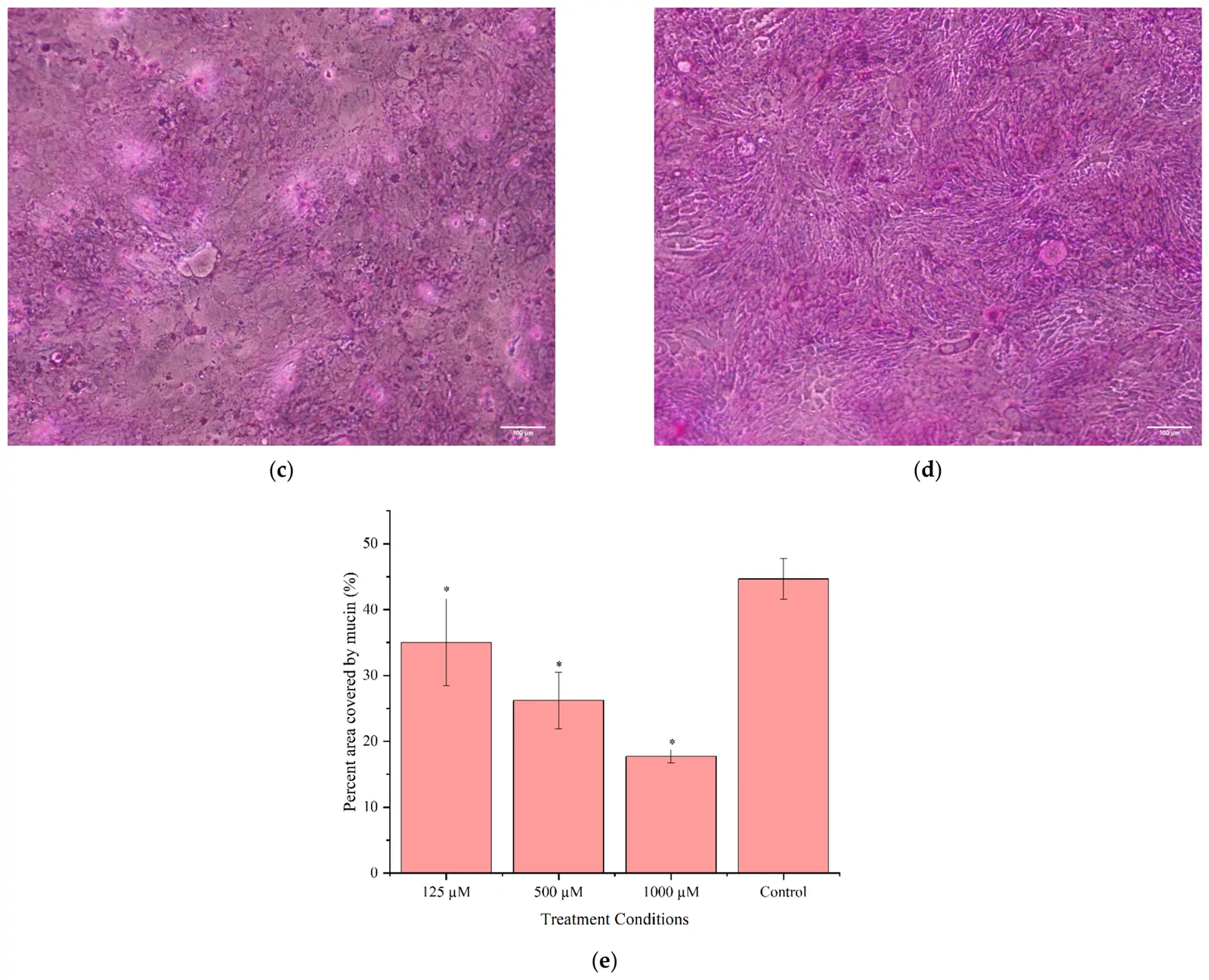
Effects of TC-ART exposure on mucin distribution in T84 monolayers. Representative images of mucin staining in T84 cells under exposure to (**a**) 125 µM, (**b**) 500 µM, and (**c**) 1000 µM TC-ART, whereas (**d**) represents controls. (**e**) Quantification of mucin-positive area expressed as percent area covered by mucin. Data are presented as mean ± SEM. Statistical significance relative to control is indicated (* *p* < 0.05) (n = 4).

Mucin expression and apical coverage were assessed in TC-ART-treated T84 monolayers using PAS staining and quantified with ImageJ/Fiji. The results show a clear dose-dependent reduction in mucin intensity and surface distribution on the monolayer, particularly at concentrations that showed a reduction in TEER and junctional gene expression. At the lowest dose, mucin levels were comparable to controls but showed a small yet significant reduction, indicating early alterations in apical surface organization (Figure 3a,b). At 500 µM, a significant decrease in mucin staining was observed, with visible thinning and patchy distribution along the apical surface (Figure 3c). At 1000 µM, mucin coverage showed a substantial loss, suggesting an impaired ability of T84 cells to secrete glycoprotein (mucin) (Figure 3d). Quantification of mucin staining confirmed a significant decline in mucin-positive area starting at 125 µM, with a further marked reduction at 500 µM and 1000 µM (Figure 3e).

### 3.3. TC-ART Slows Wound Closure in a Dose-Dependent Manner and Alters Repair-Associated Gene Expression

Because wound-healing assays were performed in non-polarized monolayers, in contrast to the polarized transwell cultures used for the permeability assay, the cellular responses reflect predominantly migratory and proliferative behavior rather than junction-dependent responses. These biological differences in model systems should be considered when comparing transcriptional responses between assays.

#### 3.3.1. Wound-Closure Rates Progressively Decline with Increasing TC-ART Exposure

**Figure 4:**
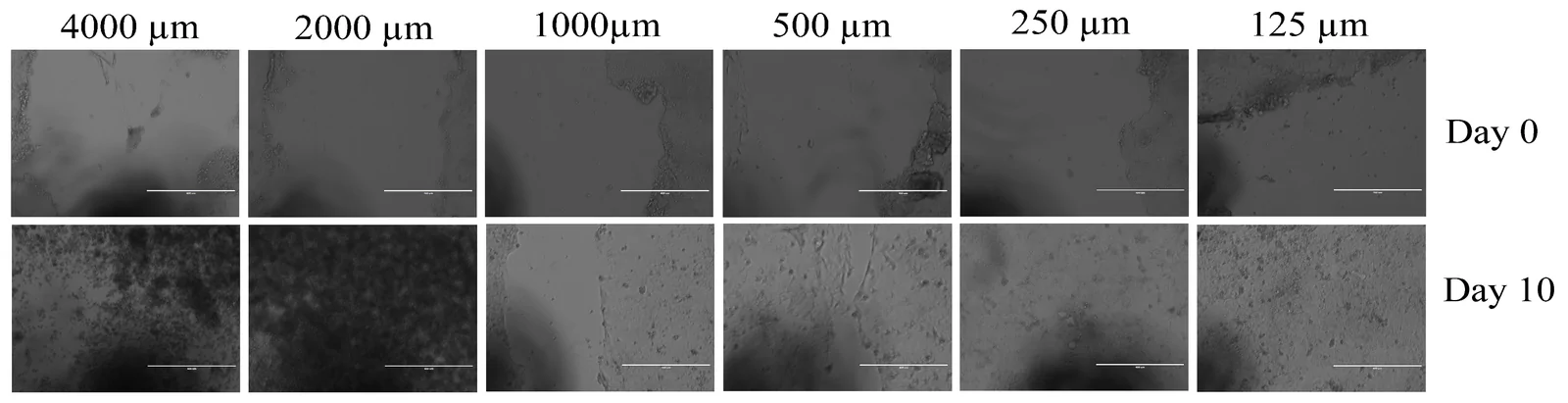
Wound-healing assay in T84 monolayers after TC-ART exposure. Representative images from a scratch wound-healing assay performed on fully confluent T84 monolayers. Images were acquired at day 0 (top panel; immediately after making scratch) and day 10 post-wounding (bottom panel) under control conditions and following treatment with increasing concentrations of TC-ART. Higher concentrations (4000 and 2000 µM) showed cytotoxic effects, whereas wound closure at 250 µM and 125 µM was comparable to control conditions. At 2000 and 4000 µM, the apparent coverage of the wound area is due to the accumulation of detached and dead cells rather than true wound closure. These images were included to illustrate the extensive cytotoxicity induced by TC-ART at high concentrations. Scale bar = 100 µm (n = 3).

The wound-healing assay demonstrated a clear concentration-dependent impairment in epithelial restitution. In control monolayers the wound area gradually decreased and was completely closed by day 7, indicating a normal ability to heal due to cell migratory and proliferation capacity (Supplementary Figure S1). At 250 µM wound healing was slower than in controls but completely closed by day 10 (Figure 4), suggesting a mild delay with preserved healing potential. However, some monolayers treated with the 125 µM concentration showed areas of cell detachment along the edges away from the wound site, which was consistent in all trials. This was likely not related to drug toxicity but could be the result of an overgrowth of cells, which sometimes causes the formation of multi-layers that cause the upper cell sheet to roll and eventually detach from the surface. At the 500 µM and 1000 µM treatments, wound closure was noticeably delayed and remained incomplete by day 10, with irregular wound margins. The highest concentrations (2000 µM and 4000 µM) caused cell death that was evident from day 1, and monolayers were almost entirely detached by day 10 or 14, consistent with LDH and MoxiGo viability data, as reported in Section 3.5. Together, these results may suggest that low and mid-doses of TC-ART interfere with migration and repair processes, whereas high doses produce cytotoxic effects that prevent wound closure.

#### 3.3.2. TC-ART Induces Dose-Dependent Alterations in Adhesion, ECM, and Repair-Associated Genes

**Figure 5:**
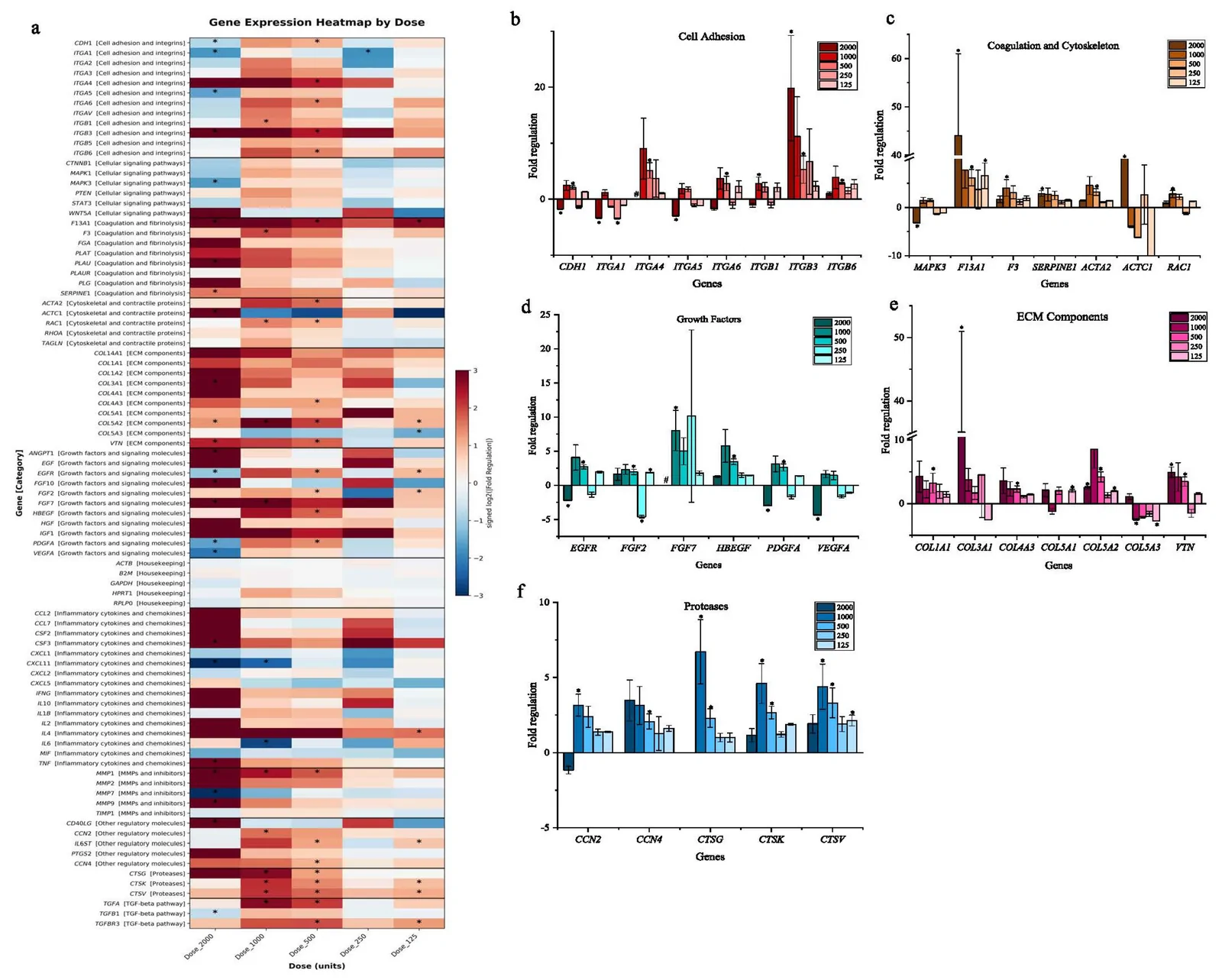
TC-ART exposure alters the expression of genes related to wound healing, specifically, those involved in the epithelial structure and extracellular matrix organization in T84 cells. (**a**) Heatmap showing fold regulation of genes following treatment with increasing concentrations of TC-ART. Gene expression values were normalized using housekeeping gene. The fold regulation was calculated as compared to the untreated controls (* *p* < 0.05). (**b**–**f**) Fold regulation of genes grouped by functional categories: (**b**) cell adhesion, (**c**) cytoskeleton organization, (**d**) growth factors, (**e**) extracellular matrix (ECM) components, and (**f**) proteases. Data are presented as mean ± SEM. Statistical significance relative to untreated control is indicated (* *p* < 0.05) (n = 3).

To delineate the molecular basis of impaired wound healing with TC-ART exposure, we examined transcriptional changes in 96 epithelial repair-associated genes, including adhesion, extracellular matrix (ECM) components, proteases, growth factors, and cytoskeletal regulators. The data show a dose-dependent multiphasic trend, with the highest number of significant changes observed at 2000 µM, 1000 µM, and 500 µM concentrations, supporting the permeability gene expression profile (Figure 5a). Overall, the lower concentrations predominantly affected pathways related to cell adhesion, ECM, and growth factor-related genes, while higher concentrations produced a broader activation of inflammatory, protease, and ECM-associated pathways. At 2000 µM, there was a maximal magnitude of upregulation of several inflammatory, proteases, and ECM modules.

The adhesion and integrin genes showed early remodeling, followed by downregulation at cytotoxic doses (Figure 5b). Up to the 1000 µM concentration several integrins (*CDH1*, *ITGA4*, *ITGA6*, *ITGB1*, *ITGB3*, and *ITGB6*) remained elevated, suggesting that cells were still attempting to re-anchor and maintain adhesion despite stress. However, at 2000 µM, where extensive detachment and cell death was observed, most adhesion-related genes (*CDH1*, *ITGA1*, and *ITGA5*) were markedly downregulated. Interestingly, ITGA4 and ITGB3 remained upregulated even at higher concentrations. As both α4 and β3 integrins have been reported in inflammatory or injury-associated adhesion contexts, their elevation here may reflect a stress-associated transcriptional response by the remaining viable cells, although this interpretation requires further validation [25]. A similar pattern was observed in MAPK3 and TGF-β gene expression. High-dose TC-ART also altered transcript levels of STAT3, MAPK1 (ERK2/1), and CTNNB1 (β-catenin) (Figure 5a). Although mRNA changes alone cannot determine pathway activation, these shifts may be consistent with the modulation of the IL-6/STAT3, EGFR–ERK, and Wnt/β-catenin networks involved in epithelial repair (Figure 5a).

Coagulation (Figure 5c), growth factor (Figure 5d), ECM (Figure 5e), proteases (Figure 5f), inflammatory cytokine (Figure 5a), and chemokine-related genes showed a dose-dependent upregulation. At higher concentrations, the widespread upregulation of these genes could be a stress-induced coping mechanism rather than a regeneration process. However, key epithelial proliferation and angiogenesis regulators like EGFR, PDGFA, and VEGFA were downregulated at higher concentrations. Similarly, CXCL11 (interferon inducible), CXCL1 (acute inflammatory signaling), and CXCL2 (inflammatory signaling) were downregulated at higher concentrations.

The morphological alterations and transcriptional profile observed in the wound-healing assay align closely with the permeability and cytokine changes described above. The increase in pro-migratory integrins and adhesion molecules at lower TC-ART (125 µM) doses aligns with the mild TEER reduction and cytokine shifts (increased IL-6 and GRO/KC). However, as concentrations increased, suppression of growth factors (*EGFPR*, *PDGFA*, *VEGF*, *FGF2*, and *HBEGF*) and adhesion genes (*CLDNs*, *ICAM*, and *NECTINs*) coincided with the loss of mucin secretion and reduced wound-healing rate.

### 3.4. Time-Dependent Transcriptional and Cytokine Responses in T84 Epithelial Monolayers Showed Impaired Wound Restitution with Short-Term TC-ART Exposure

To understand time-dependent epithelial responses and repair dynamics with TC-ART exposure we conducted a time-kinetic analysis. Initially higher concentrations (2000 µM and 4000 µM) were examined to understand the pathways involved in the cell death and the repair processes. However, extensive cytotoxicity was observed at as early as 1 h, with debris of cells accumulating around the wound margins and marked suppression of several genes (Supplementary Table S1). Based on these early toxic effects, 500 µM and 1000 µM were chosen for a subsequent time-kinetic study. No noticeable changes in wound closure or cell morphology were observed at 500 µM or 1000 µM within 24 h. However, debris from dead cells was localized near the wound edge, being more prominent at 1000 µM, suggesting localized cytotoxic stress despite overall structural continuity.

#### 3.4.1. The 500 µM Concentration of TC-ART Elicited Early Repair-Associated Gene Activation

**Figure 6:**
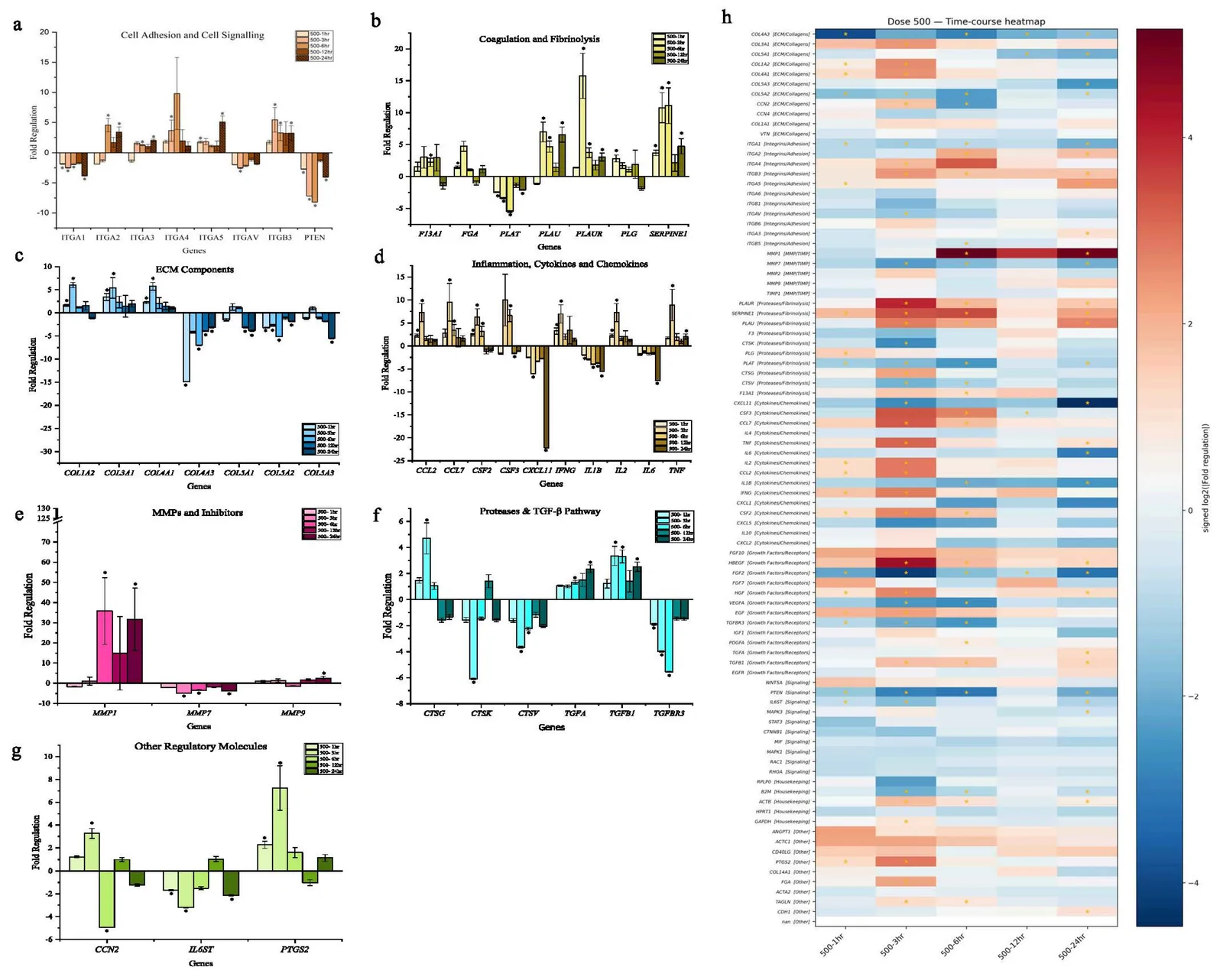
Time-dependent gene expression changes during wound healing in T84 monolayers exposed to 500 µM TC-ART. (**a**–**g**) Fold regulation of genes grouped by functional categories, including (**a**) cell adhesion and cell signaling; (**b**) coagulation and fibrinolysis; (**c**) extracellular matrix (ECM) components; (**d**) inflammation, cytokines, and chemokines; (**e**) matrix metalloproteinases and inhibitors; (**f**) proteases and tight junction-associated genes; and (**g**) other regulatory molecules. (**h**) Heatmap showing time-dependent fold regulation of genes following wound healing at 1, 3, 6, 12, and 24 for cells treated with 500 µM of TC-ART (* *p* < 0.05). Gene expression values were normalized using housekeeping gene. The fold regulation was calculated as compared to the untreated controls. Data are presented as mean ± SEM. Statistical significance relative to control is indicated (* *p* < 0.05) (n = 3).

At 500 µM, several genes involved in epithelial repair and healing processes were significantly upregulated. Integrins (*ITGA2*, *ITGA3*, *ITGA4*, *ITGA5*, and *ITGB3*) were strongly induced (Figure 6a), suggesting enhanced cell–matrix interactions and anchoring of cells for promoting migration of cells and wound healing. The downregulation of PTEN and ITGA1 may reflect alterations in motility-associated signaling, as both genes are known to participate in regulating focal adhesion dynamics and the balance between cell adhesion and migration [26,27,28]. Coagulation and fibrinolysis genes (*PLAU*, *PLAUR*, *SERPINE1*, *F13A1*, and *FGG*) were upregulated (Figure 6b), while *PLAT* remained unchanged, indicating controlled fibrinolytic activity without excessive matrix degradation. The ECM genes showed a mixed pattern, where *COL1A2*, *COL3A1*, and *COL4A1* were upregulated (Figure 6c), suggesting an increased deposition of structural components, while the proteins that are reported to aid in basement membrane stability and collagen fibril organization, *COL4A3*, *COL5A1*, *COL5A2*, and *COL5A3*, were downregulated.

Similar to the wound-healing concentration study, most cytokine-related genes were significantly altered, with *CXCL11* and *IL-6* being among the few that were downregulated (Figure 6d). *CXCL11* reduction has been noted in contexts involving changes in cell growth [29], although its relevance here requires further evaluation. *IL-6*, which is known to activate *MAPK3* and STAT3 [30], was similarly reduced, aligning with the decreased expression of these downstream genes in the concentration-dependent wound-healing dataset. The metalloproteinase profile showed a similar pattern, with *MMP1* and *MMP9* being upregulated (Figure 6e), suggesting increased ECM, whereas *MMP7* was downregulated, which aligns with the reduced mucin remodeling observed in the PAS staining. Proteases such as *CTSK*, *CTSV*, and *TGFBR3* were also decreased (Figure 6f), while some other regulatory genes showed differential regulation (Figure 6g), indicating that TC-ART exposure may influence multiple proteolytic and *TGF-β*-associated pathways, though the functional implications require further evaluation.

Overall, time-kinetic transcriptional changes showed a transient pro-repair activation at the 500 µM–3 h time point, with attenuation thereafter (see Section 3.4.3 for a comparison with 1000 µM). The kinetic data mirror with the visual changes and TEER trends showing early support then reduced resistance at 500 µM (Figure 6h). These overall changes suggest that at 500 µM, the treatment initially activates several genes involved in repair and remodeling but gradually suppresses many key pathways (Figure 6b,g,h) with time. The early increase in matrix- and adhesion-related genes may be an initial response to stress, but the continued downregulation of cytokines and signaling molecules indicates a loss of coordinate repair response with time.

#### 3.4.2. The 500 µM Concentration of TC-ART Triggered an Early Cytokine Response That Waned over Time

**Figure 7:**
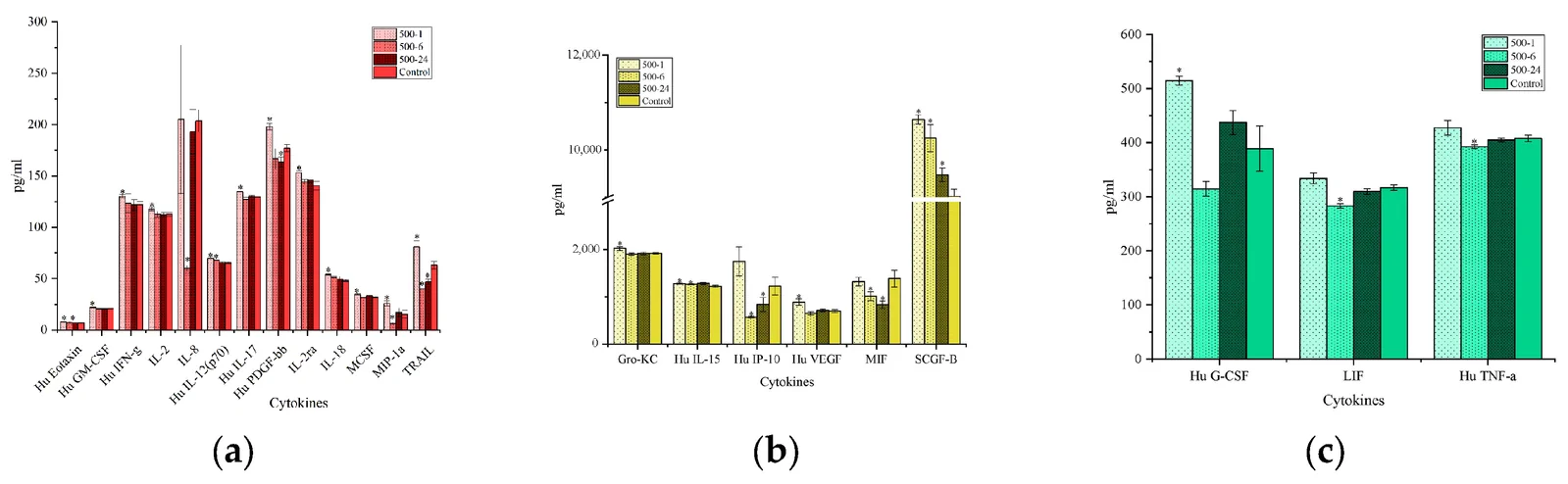
Time-dependent cytokine secretion in apical chamber media during wound-healing assay in T84 monolayers exposed to 500 µM of TC-ART. Apical cytokine levels measured at 1, 6, and 24 h post-wounding, grouped by functional category as indicated (**a**–**c**). Cytokine concentrations are expressed as pg/mL and compared to untreated controls. Data are presented as mean ± SEM. Statistical significance relative to control is indicated (* *p* < 0.05) (n = 3).

In contrast to the transcriptional patterns, cytokine secretions showed an early, rapid increase that partially normalized at 24 h. Parallel to the permeability-related cytokines IP-10, LIF, IL-9 that reduced with longer exposure, early increases were seen for IL-7, IL-6, IFN-γ, PDGF-bb, IL-17, MIP-1α, SCGF-B, IL-12(p70), IL-2, and Eotaxin, with signals highest at 1 h and declining by 24 h (Figure 7a,b). However, MIF, RANTES, LIF, IL-10, IL-9, and TRAIL showed a progressive decrease, falling below controls by 24 h. TNF-a, MCSF, GM-CSF, and VEGF showed modest changes (Figure 7a–c).

#### 3.4.3. The 1000 µM Concentration of TC-ART Produced Broad Early Transcriptional Suppression with Limited Delayed Recovery

**Figure 8:**
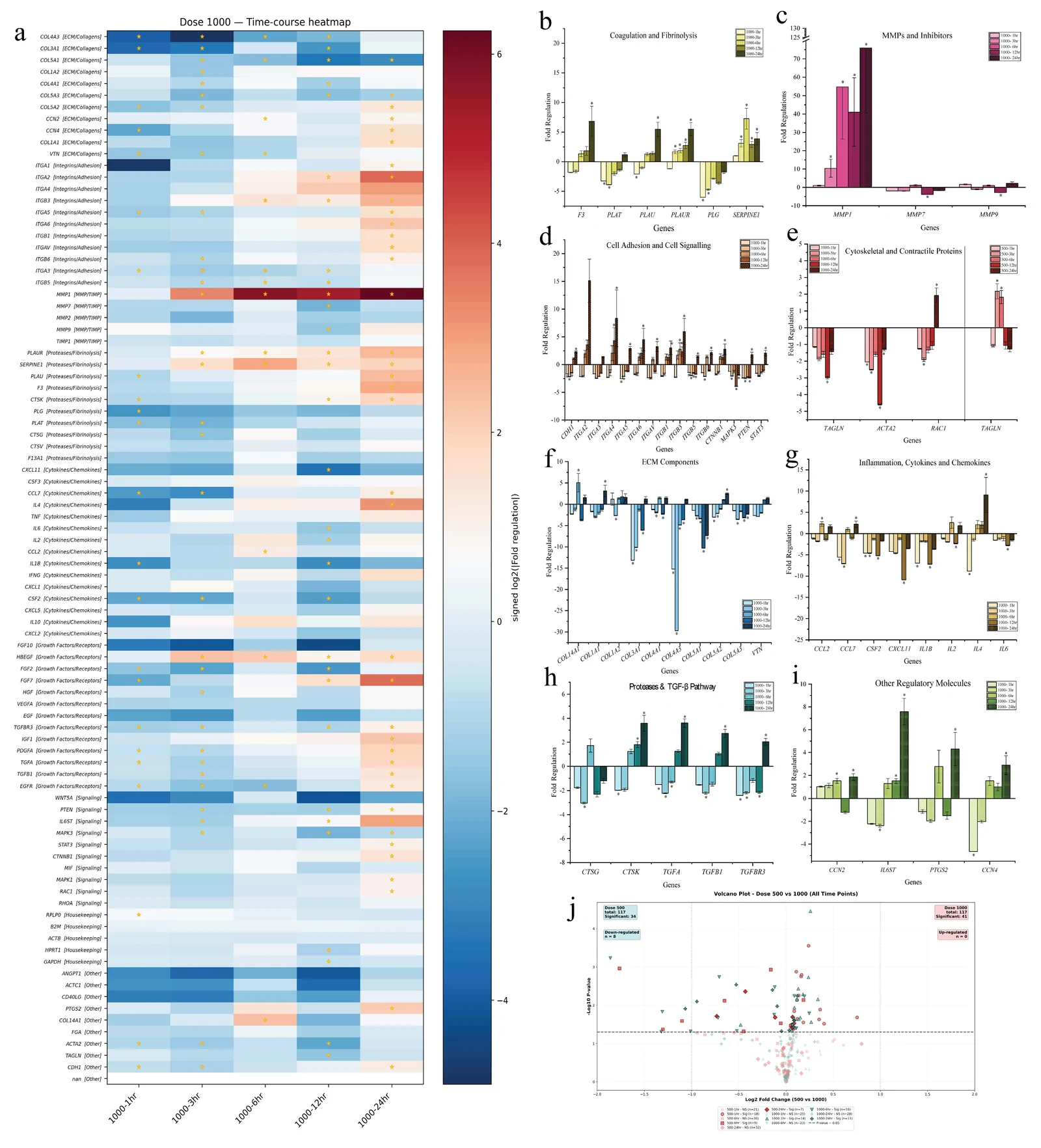
Time-dependent gene expression changes during wound healing in T84 monolayers exposed to 1000 µM of TC-ART. (**a**) Heatmap showing time-dependent fold regulation of genes following wound healing at 1, 3, 6, 12, and 24 h in cells treated with 1000 µM of TC-ART. Gene expression values were normalized using housekeeping gene. The fold regulation was calculated as compared to the untreated controls yellow star indicates *p* ≤ 0.05. (**b**–**i**) Fold regulation of genes grouped by functional categories, including (**b**) coagulation and fibrinolysis; (**c**) matrix metalloproteinases (MMPs) and inhibitors; (**d**) cell adhesion and cell signaling; (**e**) cytoskeletal and contractile proteins; (**f**) extracellular matrix (ECM) components; (**g**) inflammation, cytokines, and chemokines; (**h**) proteases and TGF-β-associated pathway genes’ (**i**) other regulatory molecules. Data are presented as mean ± SEM. Statistical significance relative to control is indicated (* *p* < 0.05) (n = 3). (**j**) Volcano plot representing up and down-regulated genes between 500 µM and 1000 µM treatments.

At 1000 µM–1 h, most genes showed strong downregulation, indicating a rapid transcriptional suppression in response to treatment (Figure 8a). Only a few genes, such as *SERPINE1*, *CCL2*, *CCN2*, and *MMP7*, showed levels near the baseline (Figure 8b,c). By 3 h, the overall expression pattern remained largely the same, with a slight upward trend observed in a few genes (*MMP1*, *SERPINE*1 and *HBEGF*), suggesting a modest shift towards partial recovery. The *SERPINE1*, *MMP1*, and *HBEGF* genes, commonly associated with extracellular matrix regulation and growth responses, showed a slight upward trend with time. MMP1 showed time-dependent upregulation. Most integrins that were significantly altered (*ITGA2*, *ITGA3*, *ITGA4*, *ITGA5*, *ITGA6*, *ITGB1*, *ITGB3*, and *ITGB6*) also showed a time-dependent increase in expression (Figure 8d), suggesting a progressive reinforcement of adhesion-related transcripts. Similarly, the coagulation and fibrinolysis genes PLAU and PLAUR also showed a time-dependent increased expression. However, PLG was downregulated, and the magnitude of this downregulation decreased over time, suggesting that PLG expression may have been gradually returning toward the baseline with extended exposure, while at 500 µM it was trending toward downregulation. The cytoskeletal and contractile genes *TAGLN* and *ACTA2* showed consistent downregulation across all time points, suggesting reduced contractile strength and cytoskeletal organization at higher concentrations, while RAC1 showed a delayed upregulation at 24 h (Figure 8e). In contrast to the 500 µM treatment, which showed up- and downregulation of ECM components, the 1000 µM concentration caused a broad suppression of ECM genes from the initial time point. *COL4A3* and *COL5A2* showed a gradual trend toward normalization at later time points, while *COL5A1* continued to decline, which may reflect early ECM disruption with only modest recovery in selected transcripts (Figure 8f). Similarly, the inflammatory cytokine and chemokine gene expression remained largely downregulated across all time points, showing an opposite trend to the 500 µM condition. Most transcripts, including *IL1B*, *IL6*, *IL2*, *CXCL11*, and *CSF2*, were consistently suppressed, indicating attenuated inflammatory gene transcription at this concentration (Figure 8g). Among the regulatory molecules, *IL6ST*, *CCN4*, *PTGS2*, *CTSK,* and *TGF-β*-related genes were transiently elevated at later time points (Figure 8h,i). Comparatively, 1000 µM showed a greater number of significantly altered genes at different time points, while 500 µM has a greater number of downregulated genes (Figure 8j).

#### 3.4.4. The 1000 µM Concentration of TC-ART Produced Early Cytokine Fluctuations Without Sustained Activation

**Figure 9:**
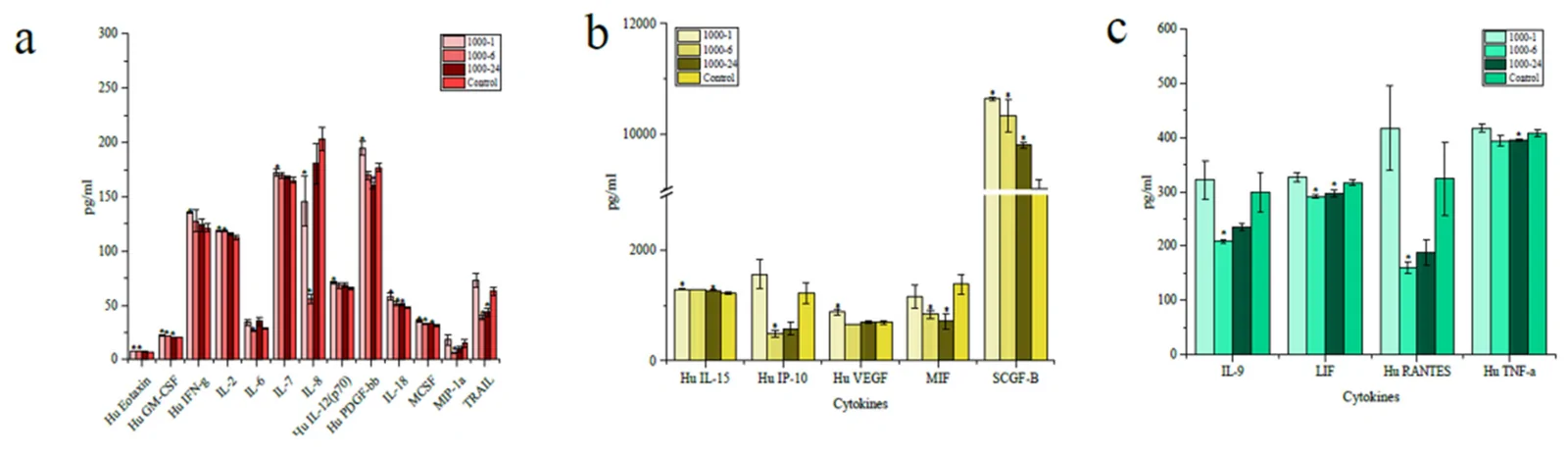
Time-dependent cytokine secretion in supernatant during wound healing in T84 monolayers exposed to 1000 µM of TC-ART. (**a**–**c**) Cytokine levels measured at 1, 6, and 24 h post-wounding, grouped by functional category as indicated. Cytokine concentrations are expressed as pg/mL and compared to untreated controls. Data are presented as mean ± SEM. Statistical significance relative to control is indicated (* *p* < 0.05) (n = 3).

The 1000 µM concentration also showed a similar magnitude of cytokine secretions. However, some of the cytokines, like IL-7 and IL-6, which showed minimal changes at 500 µM, exhibited more pronounced differences in the 1000 µM treatment (Figure 9a). Even though the gene expression profiles altered between 1000 µM and 500 µM, the cytokine profiles were broadly comparable. The 1000 µM treatment produced a short-lived increase in several cytokines during the early (1 h) phase, followed by a decline by 24 h. At 1 h, cytokines including IL-7, IL-6, IFN-γ, PDGF-BB, IL-17, MIP-1α, SCGF-B, IL-12(p70), IL-2, and Eotaxin showed higher secretion compared to controls (Figure 9b,c). By 6 h, many of them began trending downward, and by 24 h several including MIF, RANTES, LIF, IL-10, IL-9, and TRAIL were reduced below control levels (Figure 9b,c). Other cytokines, such as TNF-α, M-CSF, GM-CSF, and VEGF, showed minimal fluctuations over time.

IL-9 transcripts were downregulated at 1000 µM, coinciding with the disrupted mucin pattern observed by PAS staining. LIF and IL-15, which are commonly associated with epithelial stress and immune epithelial interactions, increased at higher concentrations. Together, these findings suggest that epithelial barrier weakening, reduced mucin coverage, and altered cytokine patterns may occur in parallel at higher TC-ART concentrations, potentially indicating a broader disruption of epithelial homeostasis.

### 3.5. TC-ART Induces Time-Dependent Cytotoxicity at Higher Concentrations

**Figure 10:**
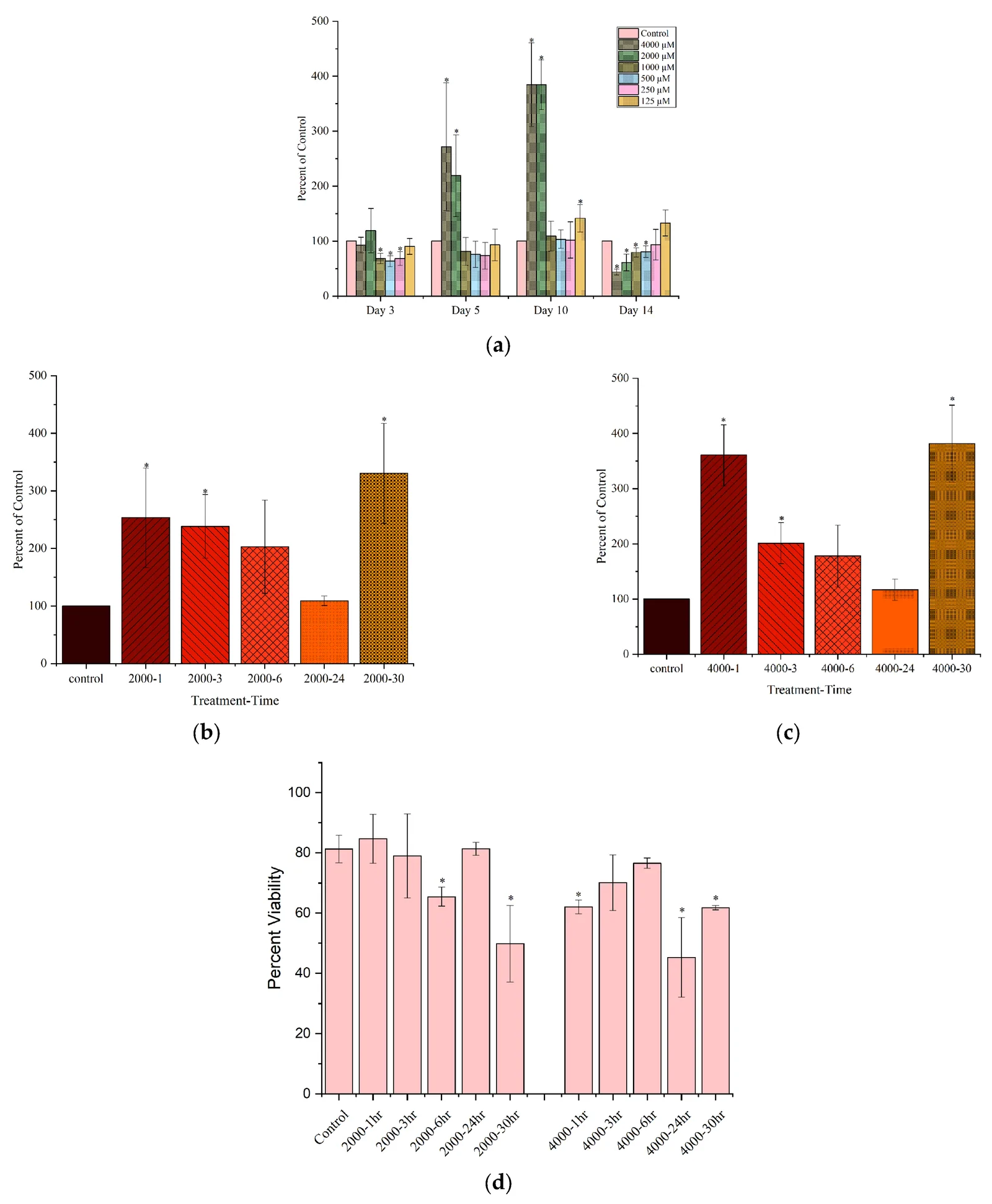
Cytotoxicity and cell viability during wound-healing assays in T84 monolayers exposed to TC-ART. (**a**) Lactate dehydrogenase (LDH) release measured during the wound-healing assay across 14 days following exposure to increasing concentrations of TC-ART (125–4000 µM). LDH levels are expressed as percentage of control. (**b**,**c**) LDH release during wound-healing time-kinetic assays following treatment with (**b**) 2000 µM and (**c**) 4000 µM of TC-ART at the indicated post-wounding time points. (**d**) Cell viability was assessed by MoxiGo during the wound-healing time-kinetic assay following exposure to 2000 µM and 4000 µM of TC-ART. Viability is presented as the measured percentage of viable cells and was not normalized to untreated controls. Data are presented as mean ± SEM. Statistical significance relative to control is indicated (* *p* < 0.05) (n = 3).

LDH release was measured from the same wells used for the wound-healing and time-kinetic assays and showed a clear concentration- and time-dependent increase at higher doses (2000 µM and 4000 µM), indicating membrane damage and reduced cell viability. In the 14-day wound-healing study, LDH release began increasing within the first few days and remained elevated through mid-experiment, with the most pronounced rise occurring between days 5 and 10 for both 2000 µM and 4000 µM (Figure 10a). By day 14, LDH levels declined, which is likely attributable to extensive cell loss and fewer viable cells available to release LDH rather than true recovery. In the shorter time-kinetic assay (1–30 h), both concentrations showed an early LDH increase between 1 and 6 h, followed by a second rise at 30 h (Figure 10b,c). Across nearly all time points, the 4000 µM group exhibited the highest LDH release, consistent with more severe membrane injury.

To complement the LDH findings cell viability was quantified using the MoxiGo assay in trypsin-detached cells from the same experiments. Viability showed a progressive decline with increasing TC-ART concentration and exposure duration, consistent with the LDH trends (Figure 10d). At 4000 µM, a marked reduction in viable cells was evident, while the 2000 µM exposure showed a moderate but sustained viability loss over time.

## 4. Discussion

The intestinal epithelium serves as a dynamic pharmacological interface but remains a highly vulnerable target for chronically administered oral therapies such as ART. TC-ART primarily targets viral enzymes but may still exert off-target effects. For example, mitochondrial dysfunction due to ART exposure has been observed in various non-infected cells [31]. Intestinal epithelial cells form a critical barrier separating commensal microbes from host tissues and serve multifactorial roles in maintaining gastrointestinal homeostasis [32]. Previous studies demonstrated that independent of HIV infection, certain ART agents can induce cytotoxic stress on the GI epithelium, mitochondrial dysfunction, and structural barrier damage [8,9,33,34]. While dolutegravir-based regimens, including combinations with abacavir and lamivudine, have shown increased gut microbial richness and reduced inflammatory activity in clinical studies [35], our previous in vivo study demonstrated intestinal permeability changes and increased pathogenic microbial species following developmental TC-ART exposure [10,11]. However, the direct cell-autonomous effects of this clinically relevant drug combination on intestinal epithelial barrier function and repair mechanisms are not studied. The present study demonstrates that exposure to TC-ART results in dose- and time-dependent changes in epithelial barrier resistance, permeability, healing abilities, and cytokine profiles. These changes were observed at sub-cytotoxic concentrations, suggesting that even non-cytotoxic levels of TC-ART can compromise epithelial integrity and may contribute to low-grade mucosal stress during prolonged exposures. Collectively, the results indicate that the epithelial responses to TC-ART are complex and evolve from early adaptive changes to structural alterations with increased doses and time of exposure. The initial reduction in TEER and downregulation of tight-junction and adhesion-related genes, such as *CLDNs*, *DSGs*, and *GJA1*, suggest a weakening of junctional continuity, and the concurrent upregulation of selected integrins and NECTINs may represent a temporary compensatory mechanism by which cells attempt to reinforce attachment and maintain barrier stability under stress. However, these adaptations appear insufficient to restore normal barrier function even at low doses, as TEER showed only a partial recovery followed by a later decline.

Among the tested concentrations the 500 µM treatment resulted in a greater number of significantly altered genes, whereas lower doses showed milder responses, while the higher dose (1000 µM) exhibited a dampened or partially suppressed transcriptional pattern. Interestingly, this mid-dose effect was not associated with obvious morphological damage, as seen in the higher dose. This molecular remodeling at 500 µM may represent an adaptive response in which cells transiently adjust transcription to maintain barrier integrity under stress. In contrast, the more pronounced transcriptional dampening at 1000 µM despite minimal immediate cytotoxicity may suggest that cells limit new gene expression under higher stress conditions, although this possibility requires further validation.

A similar trend was reflected in the wound-healing assays, where the 500 µM and 1000 µM exposures again showed patterns that broadly aligned with the permeability findings. While the magnitude of transcriptional responses differed across assays, the shorter time-kinetic study (<48 h) closely paralleled the permeability data, whereas the 14-day wound-healing assay exhibited a more distinct and pronounced dose-dependent delay in closure. This prolonged exposure likely introduced repeated cycles of stress and partial recovery, contributing to transcriptional divergence from the shorter assays. Overall, the wound-healing findings support the conclusion that prolonged TC-ART exposure disrupts epithelial migratory and regenerative capacity in a dose-dependent manner.

The concentration-dependent delay in wound closure further supports that extended TC-ART exposure disrupts epithelial repair/regeneration ability by affecting both migratory and proliferative responses. The transcriptional suppression of *STAT3*, *MAPK1/3*, and *CTNNB1* may indicate that multiple repair-related pathways [36] may be dampened, potentially limiting efficient restitution even in the absence of significant cytotoxicity; however, pathway activation cannot be inferred from gene expression alone. Reductions in components of survival- and motility-associated signaling pathways have been described in epithelial models under oxidative stress [37,38,39], where reduced IL-6/STAT3 signaling is linked to impaired restitution despite preserved viability [40]. The persistent upregulation of certain integrins, such as *ITGA4* and *ITGB3*, even at higher concentrations may reflect a stress-associated response by surviving cells attempting to maintain adhesion under adverse conditions, although this requires further evaluation [41,42]. The upregulation of *MMP1/9* and several integrins in the 500 µM time-kinetic assay may suggest an early attempt to remodel the extracellular matrix and promote migration, yet the downregulation of *COL4A3*, *COL5A1/2*, and *TGFBR3* suggests that certain components of the structural matrix may not be concurrently upregulated during this early response [43,44].

The cytokine secretion profiles further illustrate that TC-ART triggered epithelial disruption. Early increases in IL-6, IL-7, GRO/KC, and LIF indicate an initial stress and repair response, whereas the later reduction in VEGF, RANTES, and IL-9 suggests an impaired restoration of homeostasis. Interestingly, IL-2 and IL-6 were transcriptionally downregulated but showed increased secretion in the supernatants. We hypothesize that these divergences between mRNA abundance and protein levels may stem from post-transcriptional regulations. Multiple non-mutually exclusive mechanisms could contribute, such as differences in transcript and protein stability [45], continued translation of the existing mRNA pool [46], ribosome binding despite reduced transcription [47], or the release of cytokines that were translated earlier and stored in secretory vesicles [48]. In addition, cytokine abundance may have triggered a negative-feedback mechanism that could suppress further transcription, but these possibilities remain speculative and will need follow-up studies [49]. Such discrepancies have been described in other epithelial stress contexts, where transcript and protein levels do not always align [45]. While the functional consequences of these cytokine shifts are not defined here, these changes resemble patterns of low-grade inflammatory imbalance that have been described in virally suppressed individuals on long-term ART. It is important to note that mRNA transcript signatures are gaining attention as potential molecular biomarkers for the early identification of biological responses to chronic diseases, with applications in treatment monitoring, prognostic assessment, and the evaluation of drug safety [50,51,52]. Moreover, host intestinal epithelial transcript signatures, particularly genes involved in tight-junction organization, epithelial repair, and mucosal immune responses, are increasingly recognized as mechanistically informative biomarkers for assessing gastrointestinal toxicity and barrier dysfunction associated with chronic antiretroviral therapy [6].

Another key observation in this study is the dose-dependent reduction in mucin production, with patchy mucin distribution at 500 µM and 1000 µM. These changes may be consistent with the observed reduction in IL-9, a cytokine reported to regulate MUC2 expression and goblet-cell function [53]. Loss of apical mucin compromises the physical barrier that separates lumen components from the epithelial surface, making epithelial cells more susceptible to mechanical and microbial stress [54]. Furthermore, mucins can bind to and concentrate growth factors such as EGF and TGF-α near the wound site [55], thereby enhancing their availability for healing. However, TC-ART’s dose-dependent mucin reduction could limit the local availability of these protective and repair cues at the wound surface, although this requires further evaluation. This reduction in apical mucin is consistent with the decline in TEER and wound-healing ability, suggesting that TC-ART disrupts both junctional integrity and surface protection, contributing to increased barrier vulnerability. Moreover, mucin provides a hydrated matrix that facilitates epithelial cell migration during the re-epithelialization process; therefore, reduced mucin may limit efficient wound closure [50]. The mucin depletion observed in vitro aligns with our earlier in vivo findings in rat offspring exposed to TC-ART, where reduced goblet-cell density, villi atrophy, and increased intestinal permeability were observed despite indirect exposures [10]. While the present model enabled an evaluation of the direct epithelial effects of TC-ART, it did not incorporate immune cells, microbiota, or other intestinal cell types that contribute to barrier homeostasis. In addition, the observed transcriptomic changes were not validated at the protein level. Future studies incorporating human intestinal tissue and more physiologically relevant platforms, such as organ-on-chip models, will enable an evaluation of multi-cellular interactions and further strengthen the translational and regulatory relevance of these findings.

Despite these limitations, the present study provides important mechanistic insights into the plausible explanation for gastrointestinal adverse effects reported in a subset of individuals receiving TC-ART. It is worth mentioning that despite differences in exposure route and physiological context among these models (in vivo vs in vitro), the convergence of the findings supports the notion that epithelial and mucosal weakening may be an intrinsic consequence of chronic TC-ART exposure rather than merely a secondary effect of HIV infection or associated immune modulation. Collectively, these findings advance our understanding of the molecular mechanisms underlying TC-ART-induced intestinal epithelial dysfunction and provide a foundation for the development of transcript-based biomarkers and more predictive human-relevant models to improve the safety assessment of long-term antiretroviral therapies.

## 5. Conclusions

Together, these findings suggest that TC-ART exerts a broad impact on intestinal epithelial functioning, including structural, transcriptional, and secretory changes. Even at concentrations below cytotoxic thresholds, TC-ART was associated with altered coordination between junctional integrity, repair-related gene expression, and mucin secretion. Persistent exposure may therefore contribute to cumulative epithelial stress, although the long-term physiological consequences cannot be determined from this study alone. Recognizing these epithelial vulnerabilities is crucial as this highlights the need to evaluate gut health not only in the context of infection but also as an independent determinant of drug safety during lifelong therapies.

## Data Availability

The original contributions presented in this study are included in the article/supplementary material. Further inquiries can be directed to the corresponding author.

## Supplementary Materials

The following supporting information can be downloaded at: https://www.mdpi.com/article/10.3390/jox16050160/s1. Figure S1: Wound healing assay in T84 monolayer. Representative images from the healing timeline of the scratch wound: (a) control, 0 day; (b) control, day 5; (c) control, day 7. File S1: Original images of Figure 3, Figure 4 and S1. Table S1. Time and dose dependent suppression of wound healing related mRNA gene expression.
