# Supplementary material for: Tri-Combination Antiretroviral Therapy Induces Dose- and Time-Dependent Disruption of Intestinal Epithelial Barrier Function and Repair Responses in Human T84 Cells
Source: J Xenobiot. 2026 Aug 26;16(5):160. doi: 10.3390/jox16050160 (PMC13608522; doi:10.3390/jox16050160)
Supplement: Supplementary file 1 [file jox-16-00160-s001.zip › jox-4400584-Supplemental Table S1.pdf]

Supplemental Table S1. Dose and time dependent uppression of mRNA gene expression involved in wound healing

Position	Symbol	Up-Down Regulation (comparing to control group)										
		2000-1hr		2000-3hr		2000-6hr		2000-24hr		2000-30hr		4000-1hr
		Fold Regul	p-value (c	Fold Regul	p-value (c	Fold Regul	p-value (c	Fold Regul	p-value (c	Fold Regul	p-value (c	Fold Regul
A01	*ACTA2*	**-3.79**	**0.040976**	**-5.27**	**0.030693**	**-8.98**	**0.023489**	**-19.43**	**0.019987**	**-78.70**	**0.016755**	**-4.07**
A02	*ACTC1*	**-23.21**	**0.018538**	**-70.11**	**0.017257**	**-59.49**	**0.017243**	**-22.37**	**0.018574**	**-13.67**	**0.020477**	**-64.58**
A03	*ANGPT1*	**-272.98**	0.133926	**-1097.88**	0.133536	**-785.41**	0.133549	**-116.17**	0.135590	**-218.97**	0.134115	**-907.05**
A04	*CCL2*	**-815.39**	**0.016074**	**-2592.05**	**0.016024**	**-2826.55**	**0.016022**	**-245.82**	**0.016223**	**-266.85**	**0.016222**	**-1357.74**
A05	*CCL7*	**-211.91**	**0.028897**	**-1552.52**	**0.028592**	**-960.59**	**0.028675**	**-14.54**	**0.038308**	**-6.50**	**0.046304**	**-263.26**
A06	*CD40LG*	**-122.34**	**0.016457**	**-290.06**	**0.016229**	**-799.25**	**0.016163**	**-111.46**	**0.016486**	**-69.51**	**0.016897**	**-255.76**
A07	*CDH1*	1.44	0.998355	-1.65	0.221755	1.20	0.711511	1.32	0.826359	**2.39**	0.190093	1.52
A08	*COL14A1*	**-536.96**	0.287009	**-501.76**	0.287014	**-1751.95**	0.286727	**-393.73**	0.287133	**-45.05**	0.291163	**-2182.30**
A09	*COL1A1*	**-29.81**	**0.017885**	**-42.41**	**0.017320**	**-35.54**	**0.017611**	**-6.49**	**0.027853**	**-41.94**	**0.017648**	**-51.27**
A10	*COL1A2*	**-969.45**	0.316520	**-3979.53**	0.316377	**-1662.19**	0.316450	**-95.06**	0.318899	**-12.13**	0.331464	**-2318.24**
A11	*COL3A1*	**-153.32**	0.263191	**-1401.81**	0.262342	**-699.15**	0.262366	**-213.92**	0.262833	**-155.05**	0.263827	**-264.68**
A12	*COL4A1*	**-815.39**	**0.016074**	**-2592.05**	**0.016024**	**-2826.55**	**0.016022**	**-245.82**	**0.016223**	**-342.75**	**0.016176**	**-1357.74**
B01	*COL4A3*	**-815.39**	**0.016074**	**-2467.14**	**0.016026**	**-2826.55**	**0.016022**	**-51.58**	**0.017070**	**-222.54**	**0.016444**	**-211.69**
B02	*COL5A1*	**-789.32**	0.113793	**-708.93**	0.113488	**-719.72**	0.113474	**-178.92**	0.114182	**-253.83**	0.114010	**-377.78**
B03	*COL5A2*	**-47.73**	0.323236	**-61.24**	0.322429	**-42.55**	0.323656	**-45.75**	0.323366	**-40.61**	0.323875	**-44.93**
B04	*COL5A3*	**-28.39**	**0.017992**	**-35.29**	**0.017626**	**-46.57**	**0.017244**	**-31.83**	**0.018121**	**-12.99**	**0.020707**	**-24.15**
B05	*CSF2*	**-180.00**	0.101628	**-723.30**	0.101005	**-501.07**	0.101174	**-27.56**	0.106969	**-64.16**	0.103506	**-143.18**
B06	*CSF3*	**-213.07**	0.188916	**-721.57**	0.188088	**-712.63**	0.188104	**-69.18**	0.190859	**-38.21**	0.193102	**-175.90**
B07	*CCN2*	1.53	0.751114	**3.27**	**0.039389**	**3.67**	**0.029327**	**5.72**	**0.001656**	**6.08**	**0.001622**	1.11
B08	*CTNNB1*	1.08	0.734735	-1.28	0.467000	1.55	0.766038	1.56	0.734235	**3.81**	**0.024239**	-1.19
B09	*CTSG*	**-815.39**	**0.016074**	**-2592.05**	**0.016024**	**-2826.55**	**0.016022**	**-245.82**	**0.016223**	**-400.99**	**0.016140**	**-1357.74**
B10	*CTSK*	**-6.24**	**0.030785**	**-69.25**	**0.019739**	**-8.51**	**0.025193**	**-13.95**	**0.021909**	**-4.25**	**0.036172**	**-5.81**
B11	*CTSV*	1.72	0.844519	1.07	0.708080	-1.77	0.600962	**4.30**	**0.045139**	**4.03**	0.069540	1.83
B12	*CXCL1*	**-24.40**	0.103181	**-6.65**	0.132286	**-27.37**	0.234322	-1.30	0.486298	**-2.51**	0.224132	**-39.21**
C01	*CXCL11*	**-7.24**	**0.025717**	**-4.73**	**0.033364**	**-7.32**	**0.025634**	**-27.56**	**0.020238**	**-19.07**	**0.019152**	**-3.88**
C02	*CXCL2*	**-23.73**	0.201227	**-2.82**	0.309364	-1.29	0.515924	**2.62**	0.273511	1.16	0.776113	**-27.72**
C03	*CXCL5*	**-126.82**	**0.016445**	**-130.20**	**0.016440**	**-81.69**	**0.016667**	**-216.38**	**0.016250**	**-15.29**	**0.020085**	**-92.59**
C04	*EGF*	**-47.17**	0.284565	**-83.74**	0.283140	**-157.52**	0.282010	**-64.26**	0.283445	**-34.26**	0.286245	**-79.54**

C05	*EGFR*	1.55	0.317816	**-2.01**	0.098939	1.08	0.879585	1.10	0.911095	-1.15	0.437908	1.72
C06	*F13A1*	**-1427.76**	0.178574	**-468.27**	0.178871	**-582.83**	0.178793	**-430.43**	0.178919	**-702.15**	0.178727	**-2377.42**
C07	*F3*	1.12	0.945805	1.31	0.631668	1.48	0.379707	**3.05**	**0.002223**	**3.95**	**0.001460**	1.11
C08	*FGA*	**-1349.32**	0.301791	**-4289.34**	0.301681	**-4677.39**	0.301677	**-406.78**	0.302113	**-663.57**	0.301933	**-2246.80**
C09	*FGF10*	**-169.03**	**0.016339**	**-928.29**	**0.016208**	**-478.48**	**0.016210**	**-134.75**	**0.016401**	**-59.57**	**0.016914**	**-232.41**
C10	*FGF2*	**-443.56**	**0.016141**	**-2592.05**	**0.016024**	**-502.22**	**0.016196**	**-245.82**	**0.016223**	**-226.11**	**0.016426**	**-130.33**
C11	*FGF7*	**-191.77**	0.270631	**-527.72**	0.269930	**-833.32**	0.269758	**-131.22**	0.271474	**-34.58**	0.275619	**-269.70**
C12	*HBEGF*	**-5.87**	0.312793	-1.86	0.407203	**-76.95**	0.590961	**7.50**	**0.027741**	**8.84**	**0.010407**	**-3.63**
D01	*HGF*	**-815.39**	**0.016074**	**-1199.63**	**0.016073**	**-2826.55**	**0.016022**	**-245.82**	**0.016223**	**-132.34**	**0.016599**	**-1357.74**
D02	*IFNG*	**-1790.10**	0.229589	**-2339.59**	0.229555	**-6205.35**	0.229502	**-539.66**	0.229834	**-113.98**	0.231200	**-2980.76**
D03	*IGF1*	**-815.39**	**0.016074**	**-333.55**	**0.016166**	**-2826.55**	**0.016022**	**-245.82**	**0.016223**	**-42.05**	**0.017312**	**-1047.70**
D04	*IL10*	**-1221.60**	0.202742	**-497.92**	0.202995	**-207.97**	0.203758	**-39.91**	0.208353	**-23.36**	0.212903	**-569.30**
D05	*IL1B*	**-5.90**	**0.028593**	**-7.96**	**0.024560**	**-4.74**	**0.033134**	**-4.28**	**0.037438**	1.16	0.941198	**-5.23**
D06	*IL2*	**-2756.48**	0.265100	**-5090.75**	0.265076	**-9555.30**	0.265057	**-831.00**	0.265222	**-1355.58**	0.265154	**-4589.91**
D07	*IL4*	**-1257.82**	0.305356	**-1126.34**	0.305372	**-425.73**	0.305641	**-409.76**	0.305665	**-64.39**	0.308136	**-281.98**
D08	*IL6*	**-1150.84**	0.285108	**-259.93**	0.285777	**-167.81**	0.286133	**-27.59**	0.292832	**-137.83**	0.288866	**-847.33**
D09	*IL6ST*	**-6.17**	0.337212	**-16.52**	0.321284	**-6.02**	0.337260	**-4.14**	0.348774	1.20	0.526351	**-5.94**
D10	*ITGA1*	**-8.95**	0.318757	**-919.66**	0.298477	**-11.55**	0.314037	**-21.05**	0.305652	**-4.52**	0.341563	**-7.08**
D11	*ITGA2*	**-6.78**	0.121453	**-21.67**	0.098364	**-5.56**	0.129708	1.98	0.399405	**3.81**	**0.020508**	**-8.09**
D12	*ITGA3*	1.84	0.553312	-1.91	0.298923	**-6.71**	0.389456	1.38	0.980840	**2.03**	0.442917	**2.02**
E01	*ITGA4*	**-583.33**	0.119712	**-1979.08**	0.119122	**-3194.79**	0.119051	**-175.81**	0.120463	**-44.84**	0.124059	**-1713.49**
E02	*ITGA5*	**-5.14**	**0.034211**	**-10.24**	**0.023372**	**-4.67**	**0.033513**	**-4.10**	**0.038541**	**-5.68**	**0.029782**	**-2.93**
E03	*ITGA6*	1.57	0.256745	-1.90	0.136015	1.28	0.649253	**2.13**	**0.037364**	**3.13**	**0.001966**	1.35
E04	*ITGAV*	**-2.74**	0.201440	**-7.83**	0.136338	**-2.26**	0.230109	-1.11	0.453493	1.67	0.909386	**-2.79**
E05	*ITGB1*	1.80	**0.011521**	1.13	0.659191	**2.00**	**0.008813**	1.51	0.114906	**2.72**	**0.000309**	**2.09**
E06	*ITGB3*	**-272.05**	0.265266	**-330.73**	0.265566	**-134.25**	0.266590	**-11.54**	0.284102	**-14.48**	0.279381	**-437.96**
E07	*ITGB5*	**2.22**	0.504851	1.43	0.919888	1.63	0.913477	1.42	0.918906	1.98	0.661584	**2.08**
E08	*ITGB6*	**-2.63**	**0.010828**	**-6.43**	**0.000805**	**-2.13**	**0.006137**	**-7.23**	**0.000736**	**-2.29**	**0.007670**	**-2.59**
E09	*MAPK1*	-1.46	0.372626	**-2.56**	0.304224	-1.68	0.350614	**-3.10**	0.292199	**-2.42**	0.308884	-1.46
E10	*MAPK3*	1.47	0.972117	**-4.72**	0.304428	1.05	0.661548	-1.11	0.572514	-1.82	0.395951	1.67
E11	*MIF*	-1.29	0.474449	**-2.92**	**0.008246**	**-2.97**	**0.007312**	**-2.38**	**0.012878**	**-6.76**	**0.002606**	-1.64
E12	*MMP1*	**-6.58**	**0.027579**	**-5.05**	**0.032872**	-1.55	0.549907	**22.47**	**0.005994**	**52.58**	**0.000000**	**-6.60**
F01	*MMP2*	**-1463.27**	0.335018	**-4651.59**	0.334932	**-4374.25**	0.334936	**-441.13**	0.335271	**-719.61**	0.335130	**-2160.08**

F02	*MMP7*	**-2.30**	0.171689	**-3.50**	0.113974	**-4.45**	0.097575	-1.19	0.494877	**-7.95**	0.074365	**-2.82**
F03	*MMP9*	**-1934.63**	0.214644	**-592.51**	0.214849	**-2132.44**	0.214665	**-117.48**	0.216095	**-499.70**	0.214943	**-461.45**
F04	*PDGFA*	**-2.65**	0.056738	**-10.12**	**0.014153**	**-7.93**	**0.015996**	**-3.62**	**0.030319**	**-5.92**	**0.019057**	**-3.00**
F05	*PLAT*	**-481.09**	**0.016125**	**-1082.89**	**0.016080**	**-686.95**	**0.016085**	**-245.26**	**0.016223**	**-140.13**	**0.016402**	**-591.65**
F06	*PLAU*	**-22.37**	0.155978	**-38.94**	0.150925	**-10.38**	0.168687	**-4.95**	0.199258	**-5.97**	0.188540	**-21.25**
F07	*PLAUR*	**-4.02**	**0.002044**	**-4.17**	**0.001468**	-1.47	0.061079	**2.09**	**0.004153**	**2.92**	**0.000095**	**-3.60**
F08	*PLG*	**-2063.99**	0.224482	**-5229.70**	0.224428	**-1085.68**	0.224588	**-622.23**	0.224676	**-159.13**	0.225506	**-1476.50**
F09	*PTEN*	1.26	0.640633	-1.31	0.476324	-1.83	0.414312	**-9.33**	0.305348	**-4.90**	0.326303	1.17
F10	*PTGS2*	**-1165.86**	0.213149	**-3706.16**	0.212997	**-4041.44**	0.212991	**-351.47**	0.213599	**-573.35**	0.213348	**-1941.32**
F11	*RAC1*	1.21	0.554901	1.03	0.473205	-1.25	0.387676	**-2.51**	0.263108	**-2.08**	0.285394	1.49
F12	*RHOA*	1.89	0.197242	-1.39	0.409545	-1.81	0.274272	-2.00	0.210942	**-3.43**	0.114684	1.19
G01	*SERPINE1*	**-17.83**	0.357805	**-8.12**	0.366522	-1.29	0.478950	1.54	0.643138	**3.29**	0.914855	**-19.34**
G02	*STAT3*	-1.16	0.364626	**-2.46**	0.093192	-1.62	0.177160	-1.06	0.541504	**2.56**	**0.035356**	-1.23
G03	*TAGLN*	-1.63	0.166805	**-3.24**	**0.047511**	**-2.78**	0.058015	-1.13	0.468394	-1.16	0.442387	**-2.06**
G04	*TGFA*	**-2.42**	0.240711	**-7.14**	0.168853	**-3.18**	0.208563	**-2.00**	0.266531	-1.23	0.372340	**-2.54**
G05	*TGFB1*	1.69	0.901196	-1.14	0.523729	1.40	0.816175	**-8.36**	0.356709	2.00	0.812133	**2.14**
G06	*TGFBR3*	-1.53	0.162960	**-2.87**	**0.040481**	**-4.57**	**0.023503**	**-3.18**	**0.036234**	**-4.14**	**0.025864**	**-2.05**
G07	*TIMP1*	1.60	0.191208	-1.37	0.275430	-1.42	0.243631	1.23	0.676232	1.22	0.695309	1.40
G08	*TNF*	**-59.38**	**0.016923**	**-29.15**	**0.018020**	**-10.20**	**0.022253**	**-3.79**	**0.041209**	**-3.62**	**0.042054**	**-65.37**
G09	*VEGFA*	**4.17**	**0.000790**	**3.01**	**0.004792**	1.92	0.086308	-1.24	0.434320	-1.45	0.281637	**3.75**
G10	*VTN*	**-36.29**	0.195712	**-357.71**	0.190249	**-83.36**	0.192071	**-33.19**	0.200060	**-13.17**	0.208242	**-52.63**
G11	*CCN4*	**-80.65**	0.325924	**-237.52**	0.323623	**-81.83**	0.324983	**-195.15**	0.323922	**-22.16**	0.330774	**-155.77**
G12	*WNT5A*	**-176.71**	**0.016397**	**-774.57**	**0.016351**	**-440.90**	**0.016237**	**-101.27**	**0.016532**	**-250.44**	**0.016218**	**-260.06**
H01	*ACTB*	-1.16	0.438100	-1.11	0.503373	-1.07	0.605817	-1.07	0.614950	-1.15	0.441731	-1.29
H02	*B2M*	1.16	0.659370	1.11	0.794006	1.07	0.932445	1.07	0.919606	1.15	0.699162	1.29
H03	*GAPDH*	-1.50	0.238947	**-3.21**	**0.031519**	**-2.05**	0.072086	**-2.77**	**0.039810**	**-3.56**	**0.027301**	-1.55
H04	*HPRT1*	-1.38	0.384221	**-2.38**	0.177697	**-2.06**	0.211003	**-2.18**	0.199274	-1.58	0.303575	-1.82
H05	*RPLP0*	-1.21	0.485318	**-2.27**	0.107771	**-2.96**	0.072365	**-3.01**	0.072037	**-5.88**	**0.038318**	-1.88

	4000-3hr		4000-6hr		4000-24hr		4000-30hr	
p-value (co	Fold Regul	p-value (co	Fold Regul	p-value (co	Fold Regul	p-value (co	Fold Regul	p-value (comparing to control group)
**0.038476**	**-5.53**	**0.030023**	**-38.52**	**0.023785**	**-76.40**	**0.017411**	**-30.08**	**0.020992**
**0.016965**	**-72.00**	**0.017450**	**-13.55**	**0.020478**	**-26.72**	**0.018170**	**-8.78**	**0.023699**
0.133368	**-189.05**	0.134270	**-111.67**	0.135502	**-277.26**	0.133987	**-109.11**	0.135243
**0.016047**	**-557.62**	**0.016128**	**-377.43**	**0.016172**	**-215.60**	**0.016276**	**-92.40**	**0.016649**
**0.028819**	**-155.79**	**0.029064**	**-109.96**	**0.029762**	**-48.32**	**0.031345**	**-14.81**	**0.037043**
**0.016211**	**-81.67**	**0.016693**	**-70.03**	**0.016809**	**-66.35**	**0.016863**	**-26.13**	**0.018702**
0.916753	1.06	0.562144	1.00	0.508700	-1.67	0.229349	1.09	0.682821
0.286717	**-547.40**	0.287007	**-343.43**	0.287218	**-118.29**	0.289222	**-51.84**	0.291141
**0.017075**	**-17.68**	**0.019320**	**-32.31**	**0.017735**	**-23.04**	**0.018593**	**-6.78**	**0.027562**
0.316417	**-952.10**	0.316576	**-644.44**	0.316663	**-368.12**	0.316868	**-157.77**	0.317588
0.262596	**-124.42**	0.263724	**-145.92**	0.263797	**-243.18**	0.262688	**-120.24**	0.263646
**0.016047**	**-510.61**	**0.016132**	**-377.43**	**0.016172**	**-215.60**	**0.016276**	**-92.40**	**0.016649**
**0.016255**	**-304.36**	**0.016192**	**-377.43**	**0.016172**	**-215.60**	**0.016276**	**-92.40**	**0.016649**
0.113886	**-1098.02**	0.113438	**-743.21**	0.113509	**-424.54**	0.113675	**-181.95**	0.114261
0.323425	**-31.65**	0.325094	**-30.59**	0.325504	**-36.86**	0.325627	**-83.26**	0.324557
**0.018413**	**-21.66**	**0.018818**	**-43.02**	**0.017470**	**-20.80**	**0.019148**	**-44.67**	**0.017287**
0.101837	**-79.62**	0.102837	**-98.18**	0.102395	**-53.62**	0.103925	**-38.31**	0.105048
0.188954	**-87.69**	0.194849	**-77.10**	0.194008	**-165.05**	0.189115	**-13.79**	0.206584
0.797360	**2.23**	0.247822	**3.68**	0.075322	**2.64**	0.124239	**3.94**	**0.014669**
0.544643	-1.35	0.427878	-1.12	0.594648	**-4.72**	0.169159	1.07	0.975846
**0.016047**	**-557.62**	**0.016128**	**-377.43**	**0.016172**	**-215.60**	**0.016276**	**-92.40**	**0.016649**
**0.028830**	**-5.65**	**0.031314**	**-4.58**	**0.038193**	**-12.42**	**0.023532**	**-8.90**	**0.024007**
0.771683	1.10	0.735102	**2.27**	0.411654	**2.60**	0.340732	**2.78**	0.278570
0.099605	**-6.38**	0.135036	**-2.44**	0.240791	-1.39	0.436862	**-3.32**	0.181411
**0.039677**	**-5.73**	**0.029274**	**-3.66**	**0.041588**	**-9.24**	**0.023174**	**-17.94**	**0.020644**
0.198373	**-7.49**	0.225768	**-2.19**	0.369834	-1.01	0.656898	1.01	0.680470
**0.016583**	**-150.03**	**0.016389**	**-191.26**	**0.016287**	**-189.84**	**0.016298**	**-44.66**	**0.017612**
0.282774	**-19.10**	0.291969	**-37.94**	0.285727	**-85.54**	0.282603	**-25.65**	0.289752

0.141736	-1.00	0.653496	-1.36	0.255900	1.49	0.368088	**-3.25**	0.602276
0.178511	**-481.69**	0.178863	**-345.35**	0.179110	**-377.51**	0.179041	**-161.80**	0.179889
0.945073	1.68	0.232030	1.76	0.240196	1.22	0.832496	1.67	0.321942
0.301732	**-922.75**	0.301907	**-624.58**	0.302002	**-356.77**	0.302227	**-152.91**	0.303018
**0.016234**	**-100.61**	**0.016538**	**-114.63**	**0.016470**	**-133.31**	**0.016404**	**-65.91**	**0.016899**
**0.016413**	**-557.62**	**0.016128**	**-150.49**	**0.016508**	**-114.29**	**0.017179**	**-92.40**	**0.016649**
0.270216	**-103.32**	0.271462	**-110.48**	0.271457	**-142.57**	0.270960	**-111.24**	0.271366
0.337527	**-2.80**	0.358321	-1.05	0.546589	**2.88**	0.639046	**4.77**	0.189707
**0.016047**	**-557.62**	**0.016128**	**-377.43**	**0.016172**	**-215.60**	**0.016276**	**-92.40**	**0.016649**
0.229544	**-1224.19**	0.229677	**-828.61**	0.229749	**-473.32**	0.229920	**-202.86**	0.230522
**0.016056**	**-557.62**	**0.016128**	**-190.74**	**0.016322**	**-215.60**	**0.016276**	**-92.40**	**0.016649**
0.203155	**-383.62**	0.203149	**-512.73**	0.203042	**-72.17**	0.206012	**-74.79**	0.205682
**0.031104**	**-6.74**	**0.026599**	**-7.38**	**0.026549**	**-10.43**	**0.022905**	**-16.87**	**0.046268**
0.265078	**-1885.07**	0.265144	**-1275.94**	0.265180	**-728.84**	0.265265	**-312.37**	0.265564
0.305965	**-121.61**	0.307164	**-629.16**	0.305556	**-359.39**	0.305776	**-154.03**	0.306550
0.285296	**-316.95**	0.285599	**-604.76**	0.285325	**-172.79**	0.286363	**-148.05**	0.286438
0.337416	**-8.90**	0.328976	**-8.31**	0.330320	**-9.67**	0.328133	**-2.84**	0.394632
0.324660	**-22.85**	0.306097	**-12.99**	0.311358	**-30.40**	0.304883	**-13.27**	0.314240
0.114264	**-7.13**	0.118446	**-15.05**	0.103342	**-16.12**	0.101515	-1.46	0.850160
0.448765	1.07	0.692540	-1.03	0.602970	-1.35	0.474426	-1.12	0.527753
0.119118	**-703.73**	0.119366	**-418.18**	0.119533	**-230.43**	0.119914	**-116.61**	0.120947
0.053724	**-4.48**	**0.034625**	**-9.42**	**0.023423**	**-4.21**	**0.036890**	**-84.53**	**0.016758**
0.526931	-1.02	0.682367	-1.81	0.151009	**-2.11**	0.112733	1.14	0.734833
0.197458	**-3.99**	0.166374	**-3.50**	0.176817	**-2.75**	0.202554	-1.38	0.390904
**0.003215**	1.46	0.077527	1.89	0.054336	1.08	0.818185	1.36	0.281364
0.265131	**-67.79**	0.267754	**-58.35**	0.268186	**-149.82**	0.266374	**-59.40**	0.268321
0.591961	1.63	0.929351	1.75	0.796226	1.08	0.662214	1.09	0.675843
**0.003506**	**-4.00**	**0.001712**	**-3.23**	**0.002716**	**-5.62**	**0.001178**	**-4.87**	**0.001053**
0.372085	-1.50	0.367531	-1.55	0.361595	**-3.07**	0.292356	**-3.66**	0.282361
0.921358	1.13	0.710862	1.40	0.899867	-1.08	0.611453	-1.54	0.500131
**0.049065**	-1.14	0.901468	-1.12	0.521916	**-2.56**	**0.010440**	**-3.08**	**0.038595**
**0.026820**	**-5.09**	**0.034045**	-1.54	0.191703	**2.63**	**0.016829**	**16.44**	0.052323
0.334990	**-1000.68**	0.335109	**-677.33**	0.335184	**-386.90**	0.335360	**-165.82**	0.335981

0.137433	**-2.51**	0.169497	**-3.46**	0.115338	-1.31	0.407751	**-6.90**	0.081341
0.214941	**-351.64**	0.215125	**-388.45**	0.215014	**-155.75**	0.215724	**-234.69**	0.215377
**0.040167**	**-3.99**	**0.027408**	**-7.17**	**0.016676**	**-4.16**	**0.030239**	**-9.77**	**0.014757**
**0.016094**	**-212.86**	**0.016255**	**-109.09**	**0.016494**	**-215.60**	**0.016276**	**-92.40**	**0.016649**
0.156023	**-32.76**	0.152055	**-21.67**	0.156518	**-6.93**	0.182716	**-19.26**	0.158919
**0.001825**	**-4.21**	**0.001426**	**-3.57**	**0.001853**	**-2.09**	**0.008508**	1.11	0.557034
0.224521	**-908.09**	0.224597	**-466.13**	0.224781	**-319.26**	0.224991	**-233.89**	0.225218
0.606703	1.08	0.581930	-1.16	0.532171	**-5.11**	0.324406	**-4.10**	0.339757
0.213067	**-797.30**	0.213311	**-539.66**	0.213444	**-308.26**	0.213758	**-132.12**	0.214864
0.694813	1.35	0.624066	1.39	0.702412	-1.95	0.294123	**-2.45**	0.265776
0.954292	1.67	0.339943	1.15	0.816734	-1.96	0.212052	**-2.32**	0.207086
0.355762	**-6.16**	0.373308	**-5.40**	0.377154	-1.13	0.502455	1.39	0.621583
0.307928	-1.48	0.206109	**-2.50**	0.091506	**-3.31**	0.070384	-1.11	0.766294
0.098838	**-2.51**	0.070179	**-2.32**	0.087781	1.25	0.768161	-1.74	0.134012
0.228623	**-3.38**	0.203710	**-5.48**	0.178913	**-3.14**	0.211433	**-2.89**	0.217258
0.723144	1.52	0.888063	-1.06	0.556472	-1.35	0.465567	1.75	0.955129
0.074822	-1.43	0.280977	**-5.18**	**0.029179**	**-5.78**	**0.019632**	**-2.64**	0.055548
0.356695	1.15	0.844676	1.03	0.867984	-1.09	0.583868	1.05	0.908452
**0.017044**	**-23.19**	**0.018680**	**-9.09**	**0.023388**	-1.51	0.189761	**-3.62**	**0.047291**
**0.000519**	**6.00**	**0.010607**	**3.68**	**0.015499**	1.16	0.782642	-1.07	0.690600
0.193634	**-64.63**	0.192890	**-116.10**	0.192522	**-158.10**	0.190804	**-32.57**	0.196529
0.324001	**-46.79**	0.327693	**-32.90**	0.328817	**-120.63**	0.324471	**-51.30**	0.326261
**0.016209**	**-32.68**	**0.017905**	**-41.68**	**0.017428**	**-116.24**	**0.016467**	**-92.40**	**0.016649**
0.245479	-1.33	0.239903	-1.60	0.108447	-1.34	0.210494	-1.22	0.345807
0.342963	1.33	0.307446	1.60	0.085061	1.34	0.271882	1.22	0.511250
0.171519	**-2.42**	0.050084	**-2.40**	0.052679	**-2.60**	**0.046265**	**-2.55**	0.072467
0.247120	-1.78	0.268530	**-2.23**	0.191782	**-3.48**	0.126616	**-2.43**	0.174048
0.156641	-1.28	0.595633	-1.57	0.232769	**-2.67**	0.082247	**-2.96**	0.100109
