# Supplementary material for: Tri-Combination Antiretroviral Therapy Induces Dose- and Time-Dependent Disruption of Intestinal Epithelial Barrier Function and Repair Responses in Human T84 Cells
Source: J Xenobiot. 2026 Aug 26;16(5):160. doi: 10.3390/jox16050160 (PMC13608522; doi:10.3390/jox16050160)

Supplementary Materials: Tri-Combination Antiretroviral Therapy Induces Dose- and Time-Dependent Disruption of Intestinal Epithelial Barrier Function and Repair Responses in Human T84 Cells

Yaswanthi Yanamadala, Kuppan Gokulan and Sangeeta Khare

Supplemental Figure S1. Wound healing assay in T84 monolayer. Representative images from the healing timeline of the scratch wound: (a) Control 0 day, (b) Control Day 5, (c) Contril Day 7.

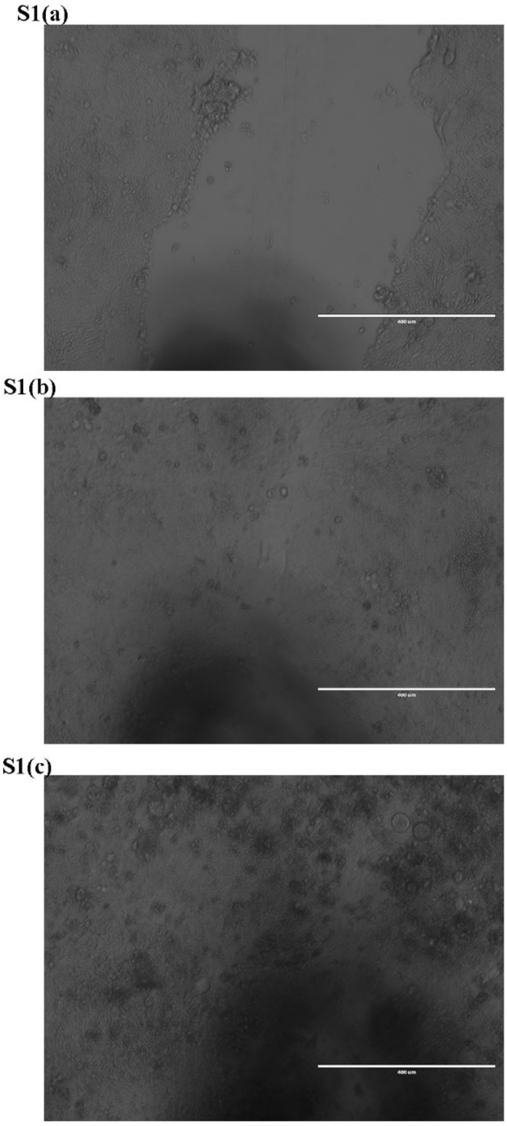

Supplement: Supplementary file 1 [file jox-16-00160-s001.zip › jox-4400584-supplementary-FigureS1.pdf]
